# Harnessing BET-Bromodomain Assisted Nuclear Import for Targeted Subcellular Localization and Enhanced Efficacy of Antisense Oligonucleotides

**DOI:** 10.1021/jacs.5c09544

**Published:** 2025-08-04

**Authors:** Disha Kashyap, Martina Cadeddu, Peter L. Oliver, Thomas A. Milne, Michael J. Booth

**Affiliations:** † Department of Chemistry, 6396 University of Oxford, Mansfield Road, Oxford OX1 3TA, U.K.; ‡ MRC Molecular Haematology Unit, MRC Weatherall Institute of Molecular Medicine, Radcliffe Department of Medicine, University of Oxford, Oxford OX3 9DS, U.K.; § MRC Nucleic Acid Therapy Accelerator, 692517 Research Complex at Harwell, Harwell Campus, Oxford OX11 0FA, U.K.; ∥ Department of Chemistry, 4919 University College London, 20 Gordon Street, London WC1H 0AJ, U.K.

## Abstract

Antisense oligonucleotides (ASOs) are a promising class of therapeutics designed to modulate gene expression. Both key mechanisms of action for ASOs operate in the nucleus: splice-switching ASOs modify pre-mRNA, processed in the nucleus, and mRNA-degrading ASOs require RNase H, an enzyme predominantly active in the nucleus. Therefore, to achieve maximal therapeutic efficacy, ASOs require efficient nuclear delivery. In this work, we have synthesized ASO conjugates for active nuclear import, by covalent conjugation with a potent and proven small-molecule nuclear importer, (+)-JQ1. (+)-JQ1 is a well-characterized high-affinity binder for members of the BET bromodomain family of proteins and was recently shown to transport cytoplasmic proteins into the nucleus. Our (+)-JQ1-ASO conjugates outperformed their unmodified counterparts for both splice-switching and mRNA knockdown in the nucleus, across different molecular targets, backbone chemistries, and cell lines. In addition, we show that the improvement in on-target efficacy correlates with increased nuclear localization of the (+)-JQ1-modified ASOs by subcellular fractionation and immunocytochemistry. Notably, we improved the performance of Oblimersen, a BCL-2 ASO drug that failed in phase-III clinical trials. (+)-JQ1-Oblimersen showed increased effectiveness in an acute myeloid leukemia cell model, showing that this therapeutic may merit re-evaluation. This work demonstrates that the covalent modification of ASOs with a small-molecule nuclear importer can significantly improve target engagement and pave the way for more effective therapeutics.

## Graphical abstract

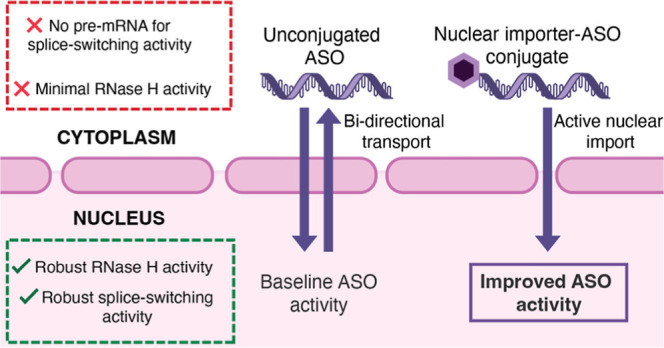

## Introduction

Nucleic acid–based therapeutics have the potential to revolutionize how we treat a wide range of diseases.^1^ Among these drugs, antisense oligonucleotides (ASOs) have attracted significant attention for their ability to provide precise control over translation.^2^ ASOs have several mechanisms of action, including RNase H-mediated degradation of mRNA bound to DNA-based ASOs, modulation of pre-mRNA processing, and steric hindrance of ribosome binding.^3^ This RNA-level intervention allows for a targeted approach to correct gene dysregulation associated with various pathological conditions, thus providing a specific and effective therapeutic strategy.

A critical aspect of ASO effectiveness consists in their ability to localize within the nucleus (Figure 1).^4^ This is because ASOs operate primarily in the nucleus, through pre-mRNA splicing and RNase H-recruitment: the only mechanisms of action featured in approved ASO drugs.^1^ ASO localization is governed by complex and dynamic intracellular trafficking mechanisms. Phosphorothioate (PS)-modified ASOs shuttle between the nucleus and cytoplasm via active transport pathways,^5^ a process influenced by factors such as backbone chemistry, protein binding partners, and cell type.^4^ While there is a pool of ASOs in the nucleus that exert their gene-modulating effects, the constant shuttling is potentially suboptimal for achieving sustained nuclear localization for maximal efficacy.

**Figure 1:**
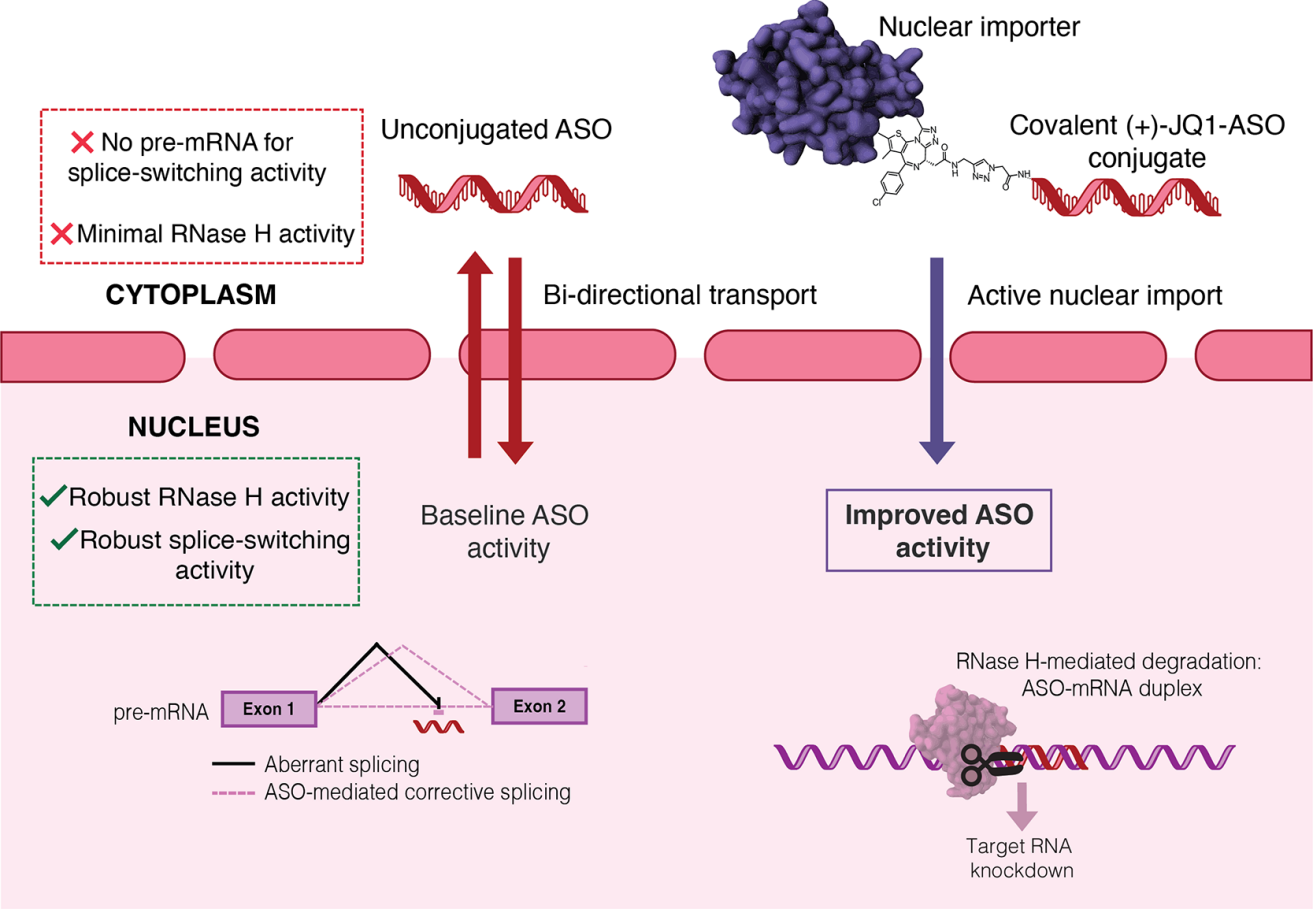
Schematic demonstrating the improved activity of (+)-JQ1-ASO conjugates over unconjugated ASOs. (+)-JQ1-ASO conjugates improve splice-modulation and RNase H-mediated knockdown through increased nuclear accumulation.

Enhancing the nuclear import and accumulation of ASOs has been postulated to significantly improve their target engagement and, consequently, their therapeutic efficacy.^6^ Previous work to improve nuclear delivery has employed nucleic acid conjugates with small molecules,^7^ aptamers,^8^ and peptides.^9^ Conjugates generated with the small molecule double-stranded DNA-binding dye Hoechst^10^ exhibited minimal and inconsistent improvements in gene-knockdown efficacy: targets with the same mechanism of action were knocked down to different degrees. Incorporating the nucleolin aptamer AS411 in a splice-switching oligonucleotide sequence has been shown to yield modest splice switching improvements.^11^ Efforts utilizing covalent ASO conjugates with nuclear localization signal (NLS) peptides—such as the canonical SV40 motif—have shown some promise.^12^ However, their broader applicability and potential toxicity remain largely untested. Furthermore, their efficacy is limited by increased endosomal entrapment, which leads to overall reduction in bioavailability.^13,14^ This demonstrates the need to explore simpler and more effective alternatives for enhanced nuclear accumulation of ASOs.

A recent study demonstrated the potential of a bifunctional compound containing the small molecule (+)-JQ1 warhead to induce the nuclear localization of cytoplasmic proteins.^15^ (+)-JQ1 is a widely studied potent binder for members of the BET bromodomain family of proteins.^16^ While most of these proteins have primary roles in the nucleus,^17,18^ they can perform secondary functions in the cytoplasm, especially in the context of cellular signaling and stress responses.^19,20^ Thus, BET bromodomain proteins display an intermediary shuttling state between the nucleus and cytoplasm.

We report a broadly applicable chemical strategy to enhance the nuclear delivery and functional activity of ASOs: covalent attachment of the bromodomain ligand, (+)-JQ1. This design exploits an entirely novel mechanism of ASO nuclear import—relying upon endogenous BET bromodomain protein trafficking. Our (+)-JQ1-ASO conjugates show superior efficacy across multiple ASO targets and diverse cell types. These (+)-JQ1-modified ASOs demonstrate higher RNase H-mediated degradation and improved splice-switching capacities—the two key mechanisms of action that feature in approved ASO drugs. Moreover, quantitative subcellular fractionation and immunocytochemistry confirmed the enhanced nuclear accumulation of the (+)-JQ1-ASO conjugates compared to the unmodified modified counterparts. We demonstrate that excess small molecule (+)-JQ1 competitively inhibits the (+)-JQ1 ASO conjugates, leading to a loss of activity and confirming a BET-dependent mechanism. Notably, this strategy significantly improves the chemosensitization performance of Oblimersen, a clinical-stage ASO with suboptimal nuclear localization—in a clinically relevant leukemia cell line. Thus, our findings establish a generalizable and modular approach to augment the nuclear localization of therapeutic oligonucleotides, with the potential to expand their clinical utility.

## Results

Throughout this work we chose to use published ASO sequences, which have been previous validated using mismatch and scrambled controls. As these sequences had been previously shown to selectively target their corresponding mRNA, the key comparative controls in our experimental validation of (+)-JQ1-ASO conjugates were ASOs without the (+)-JQ1 modification. To test the activity of (+)-JQ1-ASO conjugates, we initially chose a splice-switching ASO (SSO). The 18mer SSO we used contained a fully phosphorothioated (PS) backbone and all ribose sugars with a methyl on the 2′-position, the 2′-OMe modification (Figure 2a). This gold-standard SSO sequence was developed for the HeLa pLuc/705 cell line,^21^ which expresses a luciferase-encoding gene interrupted by a mutated β-globin intron. The mutation generates a 5′-splice site which activates a cryptic 3′-splice site, resulting in incorrect mRNA splicing and nonfunctional luciferase production (Figure 2b). The SSO binds to the mutant 5′-splice site and promotes the exclusion of the aberrant intron, restoring the pre-mRNA splicing to produce functional luciferase. Luminescence is therefore used as an indirect measure of splice-switching efficacy.

**Figure 2:**
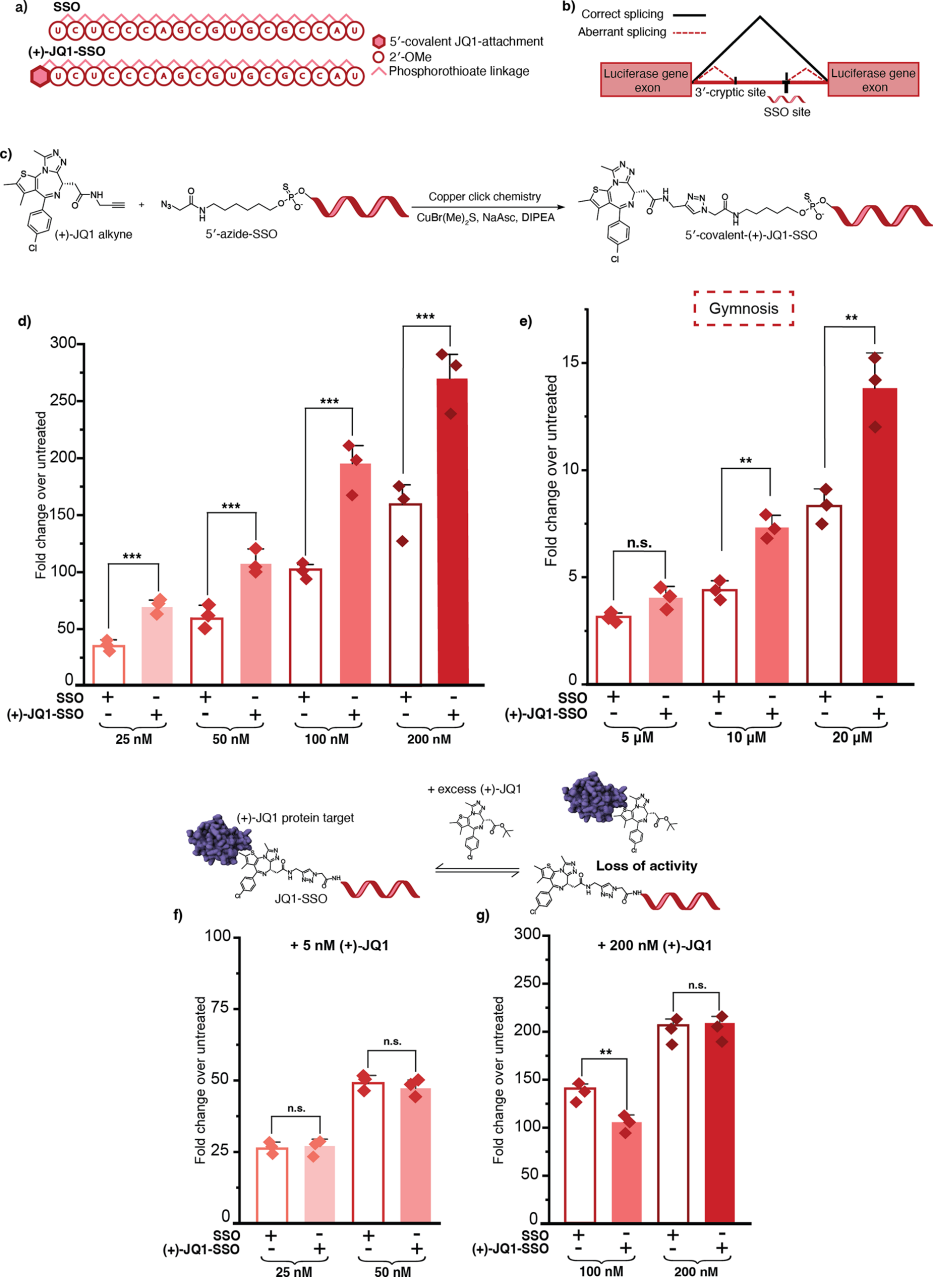
Covalent (+)-JQ1-SSO modification enhances slice-switching activity. (a) Sequence and chemical modifications for SSO used in the HeLa pLuc 705 cell line. (b) Splice-switching mechanism in HeLa pLuc 705 cells. (c) Synthesis of (+)-JQ1-SSO conjugate using copper-catalyzed click chemistry. (d) Luminescence values for SSO and (+)-JQ1-SSO activity, transfected with Lipofectamine 2000, at 24 h at concentrations indicated. In all cases, luciferase activity was measured and normalized to untreated cells. (e) Luminescence values for SSO and (+)-JQ1-SSO activity, using gymnotic delivery, at 96 h at concentrations indicated. In all cases, luciferase activity was measured and normalized to untreated cells. Competition assay between (+)-JQ1-ASO conjugate and excess small molecule, (+)-JQ1 at (f) 5 nM and (g) 200 nM. Three biological replicates are shown as diamonds for each condition (each from three technical replicates). The vertical bars represent the mean and the error bars the standard deviation. ** represents *p* < 0.05, *** represents *p* < 0.01, n.s. represents *p* values that are not significant.

To synthesize the covalent (+)-JQ1-SSO conjugate, we used copper-catalyzed click chemistry. We synthesized alkyne-modified (+)-JQ1 in two steps, starting from the commercially available (+)-JQ1 ligand. First, the boc group of (+)-JQ1 was deprotected to yield the (+)-JQ1 acid. This was followed by a hydroxybenzotriazole (HoBT) and (3-dimethylamino-propyl)-ethyl-carbodiimide hydrochloride (EDC-HCl)-mediated coupling with propargylamine, resulting in the formation of (+)-JQ1-alkyne. 5′-azide-SSO was prepared through an azidoacetic acid-*N*-hydroxysuccinimide (NHS) ester functionalization of a 5′-terminally amine-modified SSO. The final (+)-JQ1-SSO conjugate was prepared using copper-catalyzed click conjugation of the (+)-JQ1-alkyne with the 5′-azide-SSO (Figure 2c). We achieved >90% yields for all bioconjugation reactions performed and >95% purity following HPLC purification (Figures S1 and S2).

To test our (+)-JQ1-SSO conjugates in the HeLa pLuc/705 cell line, we compared their activity to the well-studied SSO without the 5′ (+)-JQ1 modification, and the intermediate azido-modified SSO. Cells were transfected with varying concentrations (25, 50, 100, and 200 nM) of either SSO (unconjugated, azido-modified, and (+)-JQ1 modified) for 24 h and quantified by luminometry, serving as a measure of splice correction and SSO efficacy. The (+)-JQ1-SSO conjugate showed 2.0-, 1.8-, 1.9-, and 1.7-fold higher splice-switching activity compared to the unconjugated and azido-modified SSO, at 25, 50, 100, and 200 nM respectively (Figures 2d and S3). Covalent addition of (+)-JQ1 resulted in little to no increase in the toxicity of the SSO at any concentration, relative to its unconjugated form—assessed through total protein production quantified by BCA (Figure S4) and CellTiter-Glo (Figure S5). To assess activity in a more physiologically relevant setting, we carried out gymnotic delivery of the (+)-JQ1-SSO conjugate, compared to its unconjugated and azido-modified counterpart, at 5, 10, and 20 μM, quantified using luminometry as above. The gymnotically delivered (+)-JQ1-SSO demonstrated significantly improved splice switching at 10 and 20 μM (Figures 2e and S6).

To confirm that the improved splice switching was induced by BET protein-mediated nuclear import rather than nonspecific binding to cellular proteins, we carried out a blockade assay with excess small molecule, (+)-JQ1. We hypothesized that excess (+)-JQ1 would saturate BET bromodomain binding sites, blocking (+)-JQ1-SSO conjugate binding and resulting in a loss of activity. At low concentrations of (+)-JQ1 (5 nM), the activity of the unmodified SSO was unaffected but the enhanced activity of the (+)-JQ1-SSO conjugate at 25 and 50 nM was effectively inhibited (Figure 2f). Consistent with the idea that the (+)-JQ1-ASO conjugate acts via an interaction with BET proteins, even higher concentrations of 200 nM (+)-JQ1 were required to inhibit the enhanced activity of the (+)-JQ1-SSO at 100 nM and 200 nM (Figures 2g and S7). Thus, we demonstrated that splice-switching ASO activity can be doubled via a specific interaction with the BET proteins for enhanced nuclear import.

Once we achieved an improvement in splice-switching activity, we wanted to test whether this approach could be extended to RNase H-mediated gene knockdown. Our test system was a 20mer ASO that targets the gold-standard knockdown target metastasis-associated lung adenocarcinoma transcript 1 (*MALAT1*), a nuclear-enriched long noncoding RNA (lncRNA).^22^ *MALAT1* plays key roles in gene regulation and metastasis in cancer, and is primarily retained within the nucleus.^23,24^ Implementing the current state-of-the-art in ASO design, this MALAT1-ASO had a gapmer design, containing a fully PS backbone with terminal wings of five 2′-methoxy-ethyl (MOE) sugar modifications (Figure 3a,b). In the gapmer design, the central region of DNA oligonucleotides is recognized by RNase H, while the flanks of 2′-modified sugars are RNase H-inactive, but enhance nuclease stability and target binding.^25^ We prepared the (+)-JQ1-MALAT1 conjugate in a similar fashion to the (+)-JQ1-SSO conjugate, by utilizing copper-catalyzed click chemistry, from the same (+)-JQ1-alkyne and a 5′-azide-modified MALAT1 gapmer. The 5′-azide MALAT1 gapmer was also prepared using azidoacetic acid-NHS ester functionalization of a 5′-terminally amine-modified MALAT1 gapmer ASO. Following copper-catalyzed click bioconjugation of the (+)-JQ1-alkyne and a 5′-azide-modified MALAT1 gapmer, the resulting (+)-JQ1-MALAT1 gapmer was produced in >90% reaction yields and >95% purity, after HPLC purification (Figures S8 and S9).

**Figure 3:**
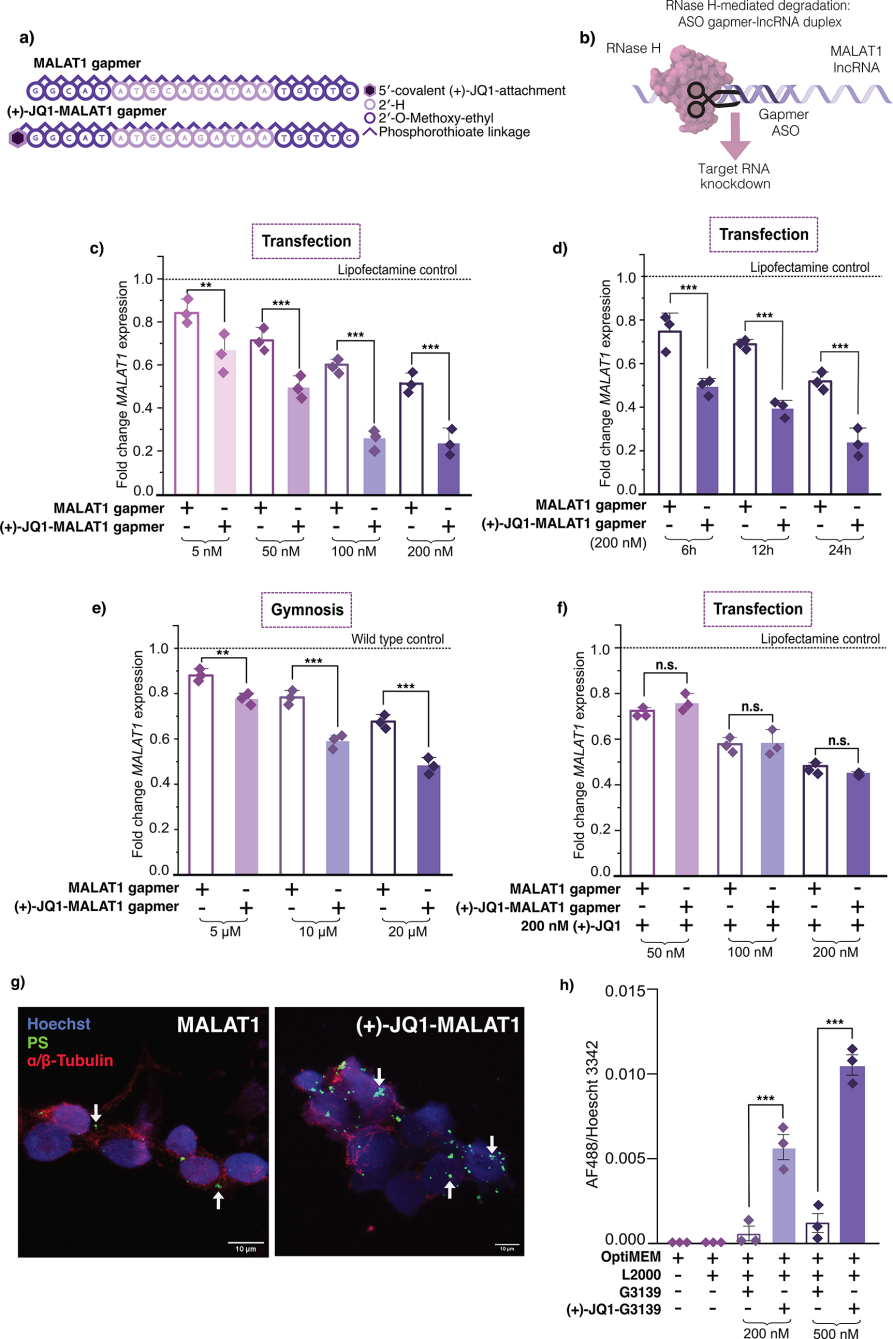
Covalent-(+)-JQ1 modification of an ASO enhances RNase H-mediated knockdown. (a) Sequence and chemical modifications for the MALAT1 gapmers used. (b) Mechanism of RNase H-mediated degradation of the lncRNA *MALAT1* by an ASO, localized in the nucleus. (c) RT-qPCR data for *MALAT1* knockdown upon Lipofectamine transfection of (+)-JQ1- and unconjugated-gapmer in HEK293T cells for 24 h at concentrations indicated. (d) RT-qPCR data for *MALAT1* knockdown upon Lipofectamine transfection of (+)-JQ1- and unconjugated-gapmer in HEK293T cells at 200 nM for 6, 12, and 24 h. (e) RT-qPCR data for *MALAT1* knockdown upon gymnosis of (+)-JQ1- and unconjugated-gapmer in HEK293T cells for 96 h at concentrations indicated. (f) *MALAT1* knockdown observed in competition assay in the presence of 200 nM (+)-JQ1. For all RT-qPCR, three biological replicates are shown as diamonds for each condition (each from three technical replicates). The vertical bars represent the mean and the error bars the standard deviation. ** represents *p* < 0.05, *** represents *p* < 0.01, n.s. represents *p* values that are not significant. (g) Representative immunocyochemistry of HEK293 cells transfected with 200 nM of unconjugated and (+)-JQ1-modified MALAT1 gapmers indicated for 24 h, using antibodies against the PS modifications (green) and α/β-tubulin (red). Arrows indicate ASO-containing puncta. Images are maximum intensity projections generated from Z-stacks; magnification 63×, scale bars as indicated. (h) Quantification of the fluorescent signal ratio between the PS immunopositive signal (AF488) within Hoechst-stained nuclei from HEK293 cells treated with the ASO concentrations as shown. Biological replicates represent random fields of view per condition. The vertical bars represent the mean and the error bars the standard deviation. *** represents *p* < 0.01.

To test the activity of the (+)-JQ1-MALAT1 gapmer conjugate, we measured *MALAT1* transcript levels using reverse transcription-quantitative polymerase chain reaction (RT-qPCR) at 24 h, comparing the knockdown to the unconjugated and azido-modified MALAT1 gapmer, normalized to the house keeping gene *GAPDH*. As with the SSO conjugate, the (+)-JQ1-MALAT1 gapmer conjugate outperformed the unconjugated and azido-modified MALAT1 gapmer at all tested concentrations. The potency was increased by 20.1%, 30.2%, 56.8%, and 54% less transcript with the (+)-JQ1 modified gapmer treatment, compared to the unmodified gapmer, at 5, 50, 100, and 200 nM respectively (Figures 3c and S10). We then tracked *MALAT1* degradation over time by assessing transcript levels through RT-qPCR at 6, 12, and 24 h following treatment. We observed a significant reduction in *MALAT1* transcript levels upon treatment with the (+)-JQ1-MALAT1 gapmer, compared to the unmodified gapmer, at all time points tested (Figure 3d). In a similar fashion to the (+)-JQ1-SSO conjugate, to assess uptake in a more clinically relevant manner, we carried out gymnotic delivery of the (+)-JQ1-MALAT1 gapmer conjugate, compared to its unconjugated and azido-modified counterpart, at 5, 10, and 20 μM, quantified using RT-qPCR as above. The gymnotically delivered (+)-JQ1-MALAT1 gapmer showed a significant increase in *MALAT1* knockdown at all tested concentrations (Figures 3e and S11).

Similar to the SSO, the enhanced activity was found to depend on specific (+)-JQ1-BET bromodomain protein interactions, as it was lost in the presence of excess quantities of small molecule (+)-JQ1. At 200 nM (+)-JQ1, the activity of the unmodified ASO was unaffected but the enhanced activity of the (+)-JQ1-MALAT1 gapmer at 50, 100, and 200 nM was completely inhibited (Figures 3f and S12). Furthermore, little to no increase in toxicity was observed for the (+)-JQ1-MALAT1 gapmer conjugate, compared to the unconjugated MALAT1 gapmer at all concentrations (Figure S13). We also expanded testing to A549 cells, a clinically relevant lung adenocarcinoma model (Figure S14). We observed a similar trend with the (+)-JQ1-modified ASO consistently outperforming its unmodified counterpart.

Next, to determine whether the addition of (+)-JQ1-modification potentiated the nuclear localization of the conjugated ASO, immunocytochemistry was carried out using an antibody raised against the PS backbone modification.^26^ HEK293 cells were transfected with either the unmodified MALAT1 gapmer or the (+)-JQ1-MALAT1 gapmer at 200 nM and 500 nM for 24 h, followed by immunostaining (Figures 3g and S15). Using the unconjugated ASO, we observed localization primarily in the perinuclear regions, as previously described for gapmers of this chemical composition.^4^ In contrast, cells transfected with (+)-JQ1-MALAT1 showed a significantly higher anti-PS signal within Hoechst-stained nuclei, compared to those treated with the unconjugated ASO (Figures 3g,h and S15), indicating enhanced nuclear localization of the JQ1-conjugated ASO. Specificity of the anti-PS immunostaining was confirmed by the absence of signal in Lipofectamine 2000-only treated cells (Figure S15).

Together, these data demonstrate that (+)-JQ1 conjugation can effectively increase both the splice-switching and gene-knockdown activity of ASOs across multiple cell lines. These functional improvements are dependent on BET-proteins, consistent with (+)-JQ1-mediated nuclear transport, and increased nuclear accumulation of the conjugated ASOs, as shown by immunocytochemistry.

While *MALAT1*-targeting antisense oligonucleotides (ASOs) are currently under evaluation for therapeutic applications, Oblimersen (G3139) represents a more advanced clinical candidate. G3139 is a first-generation 18-mer ASO with a fully PS backbone, designed to target the antiapoptotic factor *BCL-2*. It has progressed to multiple phase III clinical trials (Figure 4a,b).^27^ *BCL-2* is a crucial inhibitor of apoptosis that is overexpressed in various cancers.^28^ Despite promising phase I–II results as a sensitizer for chemotherapy, G3139 failed to show efficacy in multiple phase III trails.^29^ As this drug was well tolerated by patients,^30^ the limiting factor is likely target engagement and efficacy. Therefore, given that G3139 functions as an RNase H-active ASO, like the MALAT1 gapmer, we aimed to determine whether a (+)-JQ1-G3139 conjugate could result in a more potent drug molecule.

**Figure 4:**
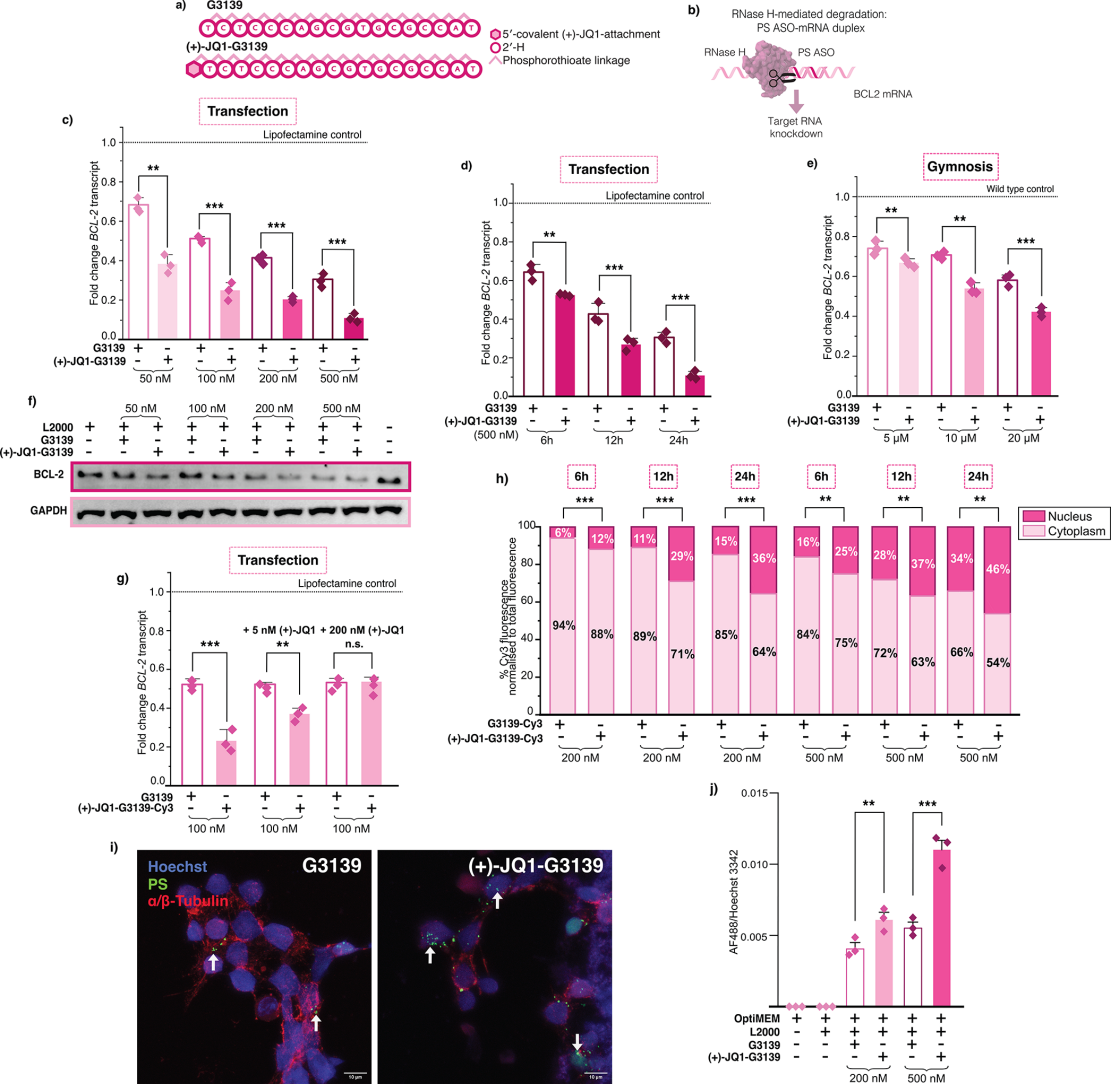
(+)-JQ1-G3139 outperformed the unconjugated G3139, a late-stage clinical ASO. (a) Sequence and chemical modifications for Oblimersen (G3139) used. (b) Mechanism of RNase H-mediated degradation of *BCL-2* mRNA by a gapmer ASO. (c) RT-qPCR data of *BCL-2* knockdown upon (+)-JQ1-G3139 and unconjugated-G3139 Lipofectamine transfection in HEK293Ts for 24 h at concentrations indicated. (d) RT-qPCR data of *BCL-2* knockdown upon (+)-JQ1-G3139 and unconjugated-G3139 Lipofectamine transfection in HEK293Ts at 500 nM at 6, 12, and 24 h. (e) RT-qPCR data of *BCL-2* knockdown upon (+)-JQ1-G3139 and unconjugated-G3139 Lipofectamine upon gymnotic delivery in HEK293Ts at 96 h at the concentrations indicated. (f) Western blot of BCL-2 levels upon treatment with G3139 and (+)-JQ1-G3139 upon transfection with Lipofectamine at 24 h at concentrations indicated. Normalized to GAPDH expression levels. (g) Reduction of enhanced BCL-2 knockdown observed in competition assay in the presence of 5 nM and 200 nM (+)-JQ1. (h) Percent Cy3 fluorescence normalized to total fluorescence in nuclear or cytoplasmic fraction upon (+)-JQ1-G3139 and unconjugated-G3139 Lipofectamine transfection in HEK293Ts for 6, 12, and 24 h at concentrations indicated. For RT-qPCR, three biological replicates are shown as diamonds for each condition (each from three technical replicates). The vertical bars represent the mean and the error bars the standard deviation. ** represents *p* < 0.05, *** represents *p* < 0.01, n.s. represents *p* values that are not significant. (i) Representative immunocyochemistry of HEK293 cells transfected with 500 nM of unconjugated and (+)-JQ1-modified G3139 for 24 h using antibodies against the PS modifications (green) and a/β-tubulin (red). Arrows indicate ASO-containing puncta. Images are maximum intensity projections generated from Z-stacks; magnification 63×, scale bars as indicated. (j) Quantification of the fluorescent signal ratio between the PS immunopositive signal (AF488) within Hoechst-stained nuclei from HEK293 cells treated with the ASO concentrations as shown. Biological replicates represent random fields of view per condition. The vertical bars represent the mean and the error bars the standard deviation. ** represents *p* < 0.05, *** represents *p* < 0.01.

We synthesized the (+)-JQ1-G3139 conjugate using the same copper-catalyzed click methodology as the previous ASOs. The 5′-azide-G3139 was synthesized from the commercially obtained 5′-amine G3139 PS ASO using azidoacetic acid-NHS ester functionalization, and the (+)-JQ1 conjugate was synthesized through a copper-catalyzed click reaction with the (+)-JQ1-alkyne (Figures S16 and S17). To assess the specificity of the (+)-JQ1 conjugate-mediated activity and evaluate any potential effects of the (+)-JQ1 moiety when conjugated to an oligonucleotide, we also included a (+)-JQ1-conjugated nontargeting phosphorothioate ASO ((+)-JQ1-NTC-ASO) control in our experimental design. The (+)-JQ1-NTC-ASO was synthesized using the same approach as described above (Figure S18). We transfected G3139, azido-G3139, (+)-JQ1-NTC ASO and (+)-JQ1-G3139 into HEK293T cells and measured the *BCL-2* transcript and protein levels, using RT-qPCR and Western blotting, respectively, after 24 h. Our (+)-JQ1-G3139 conjugate dramatically outperformed the unconjugated G3139, azido-G3139, and (+)-JQ1-NTC-ASO at all concentrations tested. On-target activity was improved by 43.9%, 51.3%, 50.9%, and 64.5% using the conjugated (+)-JQ1-G3139 compared to the unconjugated G3139 at 50, 100, 200, and 500 nM respectively (Figures 4c and S19). We also observed a concomitant marked reduction in comparative protein levels, especially at lower ASO concentrations (Figures 4d and S20). Moreover, azido-G3139 showed no significant increase in *BCL-2* knockdown compared to unmodified G3139; demonstrating that the observed effect was specific to the covalent (+)-JQ1-modification of G3139. Additionally, the (+)-JQ1-NTC-ASO showed no significant effect on *BCL-2* transcript levels; confirming that the (+)-JQ1 small molecule conjugated to an oligonucleotide alone has no impact on activity (Figure S19). We then conducted a time-course study to profile the kinetics of *BCL-2* degradation by assessing transcript levels via RT-qPCR at 6, 12, and 24 h following treatment. We observed a significant reduction in *BCL-2* transcript when using the (+)-JQ1-G3139 conjugate, compared to the unconjugated G3139, at all time points tested (Figure 4e). To assess cellular uptake in a more clinically relevant manner, we carried out gymnotic delivery of the (+)-JQ1-G3139 conjugate, compared to its unconjugated and azido-modified counterpart, and the (+)-JQ1-NTC-ASO, at 5, 10, and 20 μM, quantified using RT-qPCR as above. Gymnotically delivered (+)-JQ1-G3139 ASO showed a significant increase in knockdown compared to unmodified G3139 at all tested concentrations at the RNA level (Figure 4f). Furthermore, as with transfection, gymnotically delivered azido-G3139 showed no significant difference in *BCL-2* knockdown compared to the unconjugated G3139 and the (+)-JQ1-NTC-ASO showed little/no effect on *BCL-2* transcript levels (Figure S21).

We then conducted a blockade assay using excess small molecule (+)-JQ1, similar to the approach used with the other conjugates. As expected, at both the 5 and 200 nM dose of small molecule, (+)-JQ1, the activity of unmodified G3139 was unaffected. However, at 5 nM (+)-JQ1 the enhanced activity of (+)-JQ1-G3139 at 100 nM showed a significant reduction, and at 200 nM (+)-JQ1, the enhanced activity of (+)-JQ1-G3139 at 100 nM was fully inhibited (Figure 4g). As observed with the previous (+)-JQ1 conjugates, little to no increase in cellular toxicity was observed for (+)-JQ1-G3139, compared to the unconjugated ASO (Figure S22).

To confirm that the enhanced ASO activity was a result of increased nuclear accumulation, we quantified the levels of G3139 and (+)-JQ1-G3139 conjugate within the nucleus and cytoplasm using subcellular fractionation. To enable simple fluorescent-based detection of ASO localization, we synthesized a (+)-JQ1-G3139-Cy3 conjugate. The (+)-JQ1-G3139-Cy3 conjugate was synthesized as above, from the commercially available 5′-terminal amine- and 3′-terminal Cy3-modified G3139 PS ASO. A 5′-azide was installed using azidoacetic acid-NHS ester functionalization, and the final (+)-JQ1-G3139-Cy3 conjugate was synthesized through a copper-catalyzed click reaction of the 5′-azide-G3139–3′-Cy3 with the (+)-JQ1-alkyne (Figures S23 and S24). We first verified, using RT-qPCR, that the addition of the Cy3 moiety at the 3′-end of G3139 or (+)-JQ1-G3139 did not alter the knockdown activity observed above (Figure S25). We then transfected the cells with either G3139-Cy3 or (+)-JQ1-G3139-Cy3 for 6, 12, and 24 h, followed by subcellular fractionation (Figure S26), and measured the fluorescence intensity of the cytoplasmic and nuclear fractions via a plate reader. We observed a significant increase in nuclear accumulation of the (+)-JQ1-G3139-Cy3 conjugate compared to its unmodified counterpart, for all time points, at both 200 nM and 500 nM (Figures 4h and S27). As we observed higher concentrations of the (+)-JQ1-modified G3139 in the nuclear fraction compared to unmodified G3139, this indicated that the enhanced nuclear accumulation was driven by the covalent (+)-JQ1 modification. Furthermore, the nuclear and cytoplasmic distribution of G3139 and (+)-JQ1-G3139 at 200 nM and 500 nM was consistent with the activity trends measured at the protein and RNA level. To corroborate these findings—which relied on fluorescently labeled ASOs—we performed anti-PS immunocytochemistry using (+)-JQ1-G3139 and unconjugated G3139 without fluorescent tags following 24 h transfection at 200 nM and 500 nM (Figures 4i,j and S28). In line with the results from MALAT1-targeted staining, we observed a significant increase in the nuclear-localized signal for the (+)-JQ1-G3139 conjugate compared to the unconjugated G3139 ASO (Figures 4j and S28). As previously shown, specificity of the anti-PS immunostaining was confirmed by the absence of signal in Lipofectamine 2000-treated cells (Figure S28).

To test our nuclear localization strategy in a more clinically relevant model, we evaluated the activity of the (+)-JQ1-G3139 conjugate in THP-1 cells, an acute myeloid leukemia (AML) cell line. G3139 previously progressed to phase III clinical trials as a chemosensitizer in AML for cytarabine-dosing regimens^29^ (Figure 5a), highlighting its therapeutic potential. Testing the conjugate in this context allowed us to assess the potential of JQ1-mediated ASO nuclear transport in a disease-relevant setting. We delivered G3139, azido-G3139, (+)-JQ1-G3139, and (+)-JQ1-NTC-ASO into THP-1 cells using electroporation and measured the BCL-2 transcript levels using RT-qPCR after 48 h. Our (+)-JQ1-G3139 conjugate dramatically outperformed the unconjugated G3139 at all concentrations tested. We measured 23.6%, 35.8%, and 48.3% less transcript using the modified (+)-JQ1-G3139, compared to the unconjugated G3139, at 200 nM, 500 nM and 1 μM respectively (Figure 5b). As previously observed, azido-G3139 showed no significant difference in BCL-2 knockdown compared to the unconjugated G3139 and the (+)-JQ1-NTC-ASO showed little/no effect on BCL-2 transcript levels; demonstrating again that increased efficacy was due to the covalent (+)-JQ1 modification (Figure S29). Furthermore, upon conducting a blockade assay using excess small molecule (+)-JQ1, similar to the approach used with G3139 in HEK293Ts, we obtained similar results. As expected, at both the 5 and 200 nM (+)-JQ1, the activity of unmodified G3139 was unaffected. However, at an excess of 5 nM (+)-JQ1, the enhanced activity of (+)-JQ1-G3139 at 1 μM showed a significant reduction, and at 200 nM (+)-JQ1, the enhanced activity of (+)-JQ1-G3139 was fully lost (Figure 5c). This confirmed that the mechanism of action for the (+)-JQ1-mediated ASO nuclear transport could be extended to THP-1 cells. We then sought to confirm that we achieved protein level knockdown by measuring BCL-2 protein levels after a second round of electroporation, with Western blotting at 96 h (Figures 5d, S30 and S31). We observed a marked reduction in protein levels at all tested concentrations, in line with the trend observed at the RNA level. Finally, to evaluate the chemosensitizing potential of (+)-JQ1-G3139, we performed a CellTiter-Glo assay. THP-1 cells were treated with G3139 alone and in combination with cytarabine to establish a baseline response, followed by treatment with (+)-JQ1-G3139 alone and in combination with cytarabine (Figure 5e). Notably, (+)-JQ1-G3139 treatment resulted in a significantly greater reduction in cell viability compared to the unconjugated G3139, both as a single agent and in combination with cytarabine.

**Figure 5:**
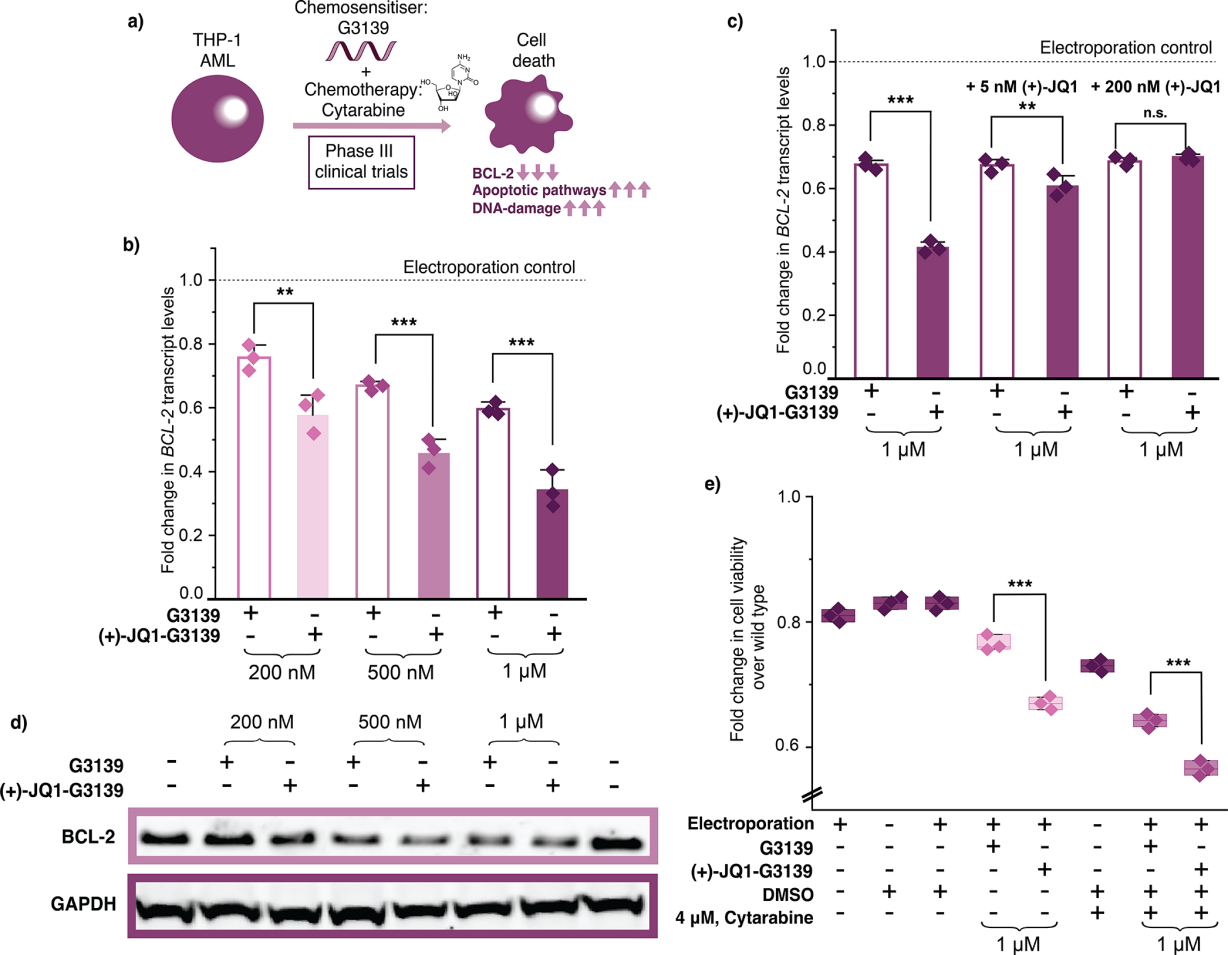
(+)-JQ1-G3139 shows enhanced knockdown and chemosensitization in THP-1 cells, a clinically relevant model for AML. (a) Schematic for G3139 mechanism of action in the THP-1 AML cell line. (b) RT-qPCR data of *BCL-2* knockdown upon (+)-JQ1-G3139 and unconjugated-G3139 upon electroporation for 48 h at concentrations indicated. (c) Reduction of enhanced *BCL-2* knockdown observed in competition assay in the presence of 5 nM and 200 nM (+)-JQ1. (d) Western blot of BCL-2 levels following treatment with G3139 and (+)-JQ1-G3139 upon two rounds of electroporation at 96 h at concentrations indicated. Normalized to GAPDH expression levels. (e) Cell viability upon treatment with G3139 and (+)-JQ1-G3139 via electroporation for 48 h as single agents or in combination with cytarabine, at concentrations indicated. For RT-qPCR, three biological replicates are shown as diamonds for each condition (each from three technical replicates). The vertical bars represent the mean and the error bars the standard deviation. ** represents *p* < 0.05, *** represents *p* < 0.01, n.s. represents *p* values that are not significant.

## Discussion

Our work establishes a novel, simple, and broadly applicable chemical strategy to improve the nuclear localization and enhance the potency of ASOs via covalent conjugation with a small-molecule BET-bromodomain ligand, (+)-JQ1. This modular approach significantly improves ASO efficacy across multiple targets, diverse backbone chemistries, transfection and gymnotic delivery, and both major ASO mechanisms of action—RNase H-mediated degradation and splice modulation. The ASO chemistries and mechanisms of action we employ feature in several clinically approved nucleic acid therapeutics, highlighting the translational potential of our work. In vivo studies will be required to further validate the clinical translation of this approach.

By demonstrating that conjugation to (+)-JQ1 enhances nuclear accumulation of ASOs in a BET bromodomain-dependent manner, we build upon the previous literature surrounding the development of bromodomain inhibitor, (+)-JQ1, as a nuclear importer.

Our data underscores the importance of precisely targeting therapeutic agents to their site of action. By inducing ASO enrichment in the nucleus, we have improved their functional efficacy and therapeutic potential. In this way, we have shown that nuclear localization is crucial for maximizing the therapeutic potential of ASOs for splice switching and RNase H-mediated knockdown. Notably, we apply our strategy to Oblimersen, an extensively studied ASO targeting BCL-2, which has been taken to Phase III clinical trials multiple times but shown limited efficacy. Our approach, i.e. covalent conjugation with (+)-JQ1, dramatically improves Oblimersen’s nuclear localization, functional BCL-2 knockdown, and chemosensitization efficacy in a clinically relevant AML model. Our approach might also have the potential to transform other suboptimal ASO drugs into more effective therapeutics.

The modular nature of this technology opens exciting possibilities for future applications. By demonstrating that small molecules can be potent effectors of ASO subcellular compartmentalization and thus, functionality, we pave the way for the development of advanced therapeutic strategies through small molecule conjugation. The integration of small molecules to manipulate cellular environments and target sites offers a promising approach to enhance therapeutic outcomes of nucleic acid–based therapeutics.

While our study focuses on the proof-of-concept application of (+)-JQ1 as a nuclear localization enhancer, we recognize that BET bromodomain ligands like JQ1 can have broader transcriptomic effects due to their role as epigenetic modulators. Moreover, certain ASO gapmer chemistries can disrupt nuclear organization and RNA processing, highlighting the need for careful design to minimize off-target effects.^31^ Future work will explore alternative ligands or delivery strategies to minimize off-target transcriptional consequences while retaining nuclear targeting efficiency. Additionally, it will be important to elucidate the precise mechanism of nuclear accumulation, including whether it occurs via active transport or passive retention. Linker architecture may also impact cellular uptake, nuclear import, or conjugate orientation. In this study, we employed a minimal azido-acetic acid linker for its simplicity and high-yielding conjugation, but future iterations will explore linker length, polarity, and flexibility to further optimize intracellular trafficking and nuclear localization.

Our work aligns with the burgeoning field of bifunctional molecules, which combines distinct functionalities into a single entity.^32^ This intersection with bifunctional technologies allows our approach to be integrated into the broader context of therapeutic innovation, by leveraging the synergies between small-molecule ligands and nucleic acid–based therapeutics.

In summary, our study provides a compelling demonstration of how small molecules can be harnessed to enhance ASO activity through improved nuclear localization, for multiple targets and mechanisms of action. The modular nature of this technology, combined with its compatibility with emerging bifunctional modalities, positions it as a promising platform for future therapeutic design.

## Supplementary Material



## Data Availability

All the data generated in this study are available within the article, the Supporting Information, and figures. Source data will be made available on Zenodo upon acceptance of the manuscript.

## Notes

Detailed methods for all organic syntheses, oligonucleotide functionalization and purification, and biological experiments; full characterization data including NMR, HR-MS, and oligonucleotide HPLC and MS; ASO and qPCR primers sequences. Figures S1–S31 contain synthetic routes for ASO functionalization, analytical validation, ASO activity assays such as qPCR and Western blotting, cytotoxicity, immunocytochemistry and microscopy, and subcellular localization studies (PDF)
