# Supplementary material for: Harnessing BET-Bromodomain Assisted Nuclear Import for Targeted Subcellular Localization and Enhanced Efficacy of Antisense Oligonucleotides
Source: J Am Chem Soc. 2025 Aug 4;147(32):29478–88. doi: 10.1021/jacs.5c09544 (PMC12356583; doi:10.1021/jacs.5c09544)
Supplement: Supplementary file 1 [file ja5c09544_si_001.pdf]

Supplementary Information for:

Harnessing BET-bromodomain assisted nuclear import for targeted subcellular localization and enhanced efficacy of antisense oligonucleotides

Disha Kashyap[1,2], Martina Cadeddu[3], Peter L. Oliver[3], Thomas A. Milne[2*], Michael J. Booth[1,4*]

[1]Department of Chemistry, University of Oxford, Mansfield Road, Oxford, OX1 3TA, U.K.
[2]MRC Molecular Haematology Unit, MRC Weatherall Institute of Molecular Medicine, Radcliffe Department of Medicine, University of Oxford, Oxford, OX3 9DS, U.K.
[3]MRC Nucleic Acid Therapy Accelerator, Research Complex at Harwell, Harwell Campus, Oxford, OX11 0FA, U.K.
[4]Department of Chemistry, University College London, 20 Gordon Street, London, WC1H 0AJ, U.K.

*Correspondence: thomas.milne@imm.ox.ac.uk, m.j.booth@ucl.ac.uk

Table of Contents

1. **Materials and Methods**………………………………………………………………………………………..2
   1.1. Organic Synthesis
      1.1.1. General information about reagents, purification, and characterisation
      1.1.2. (S)-2-(4-(4-chlorophenyl)-2,3,9-trimethyl-6H-thieno[3,2-f][1,2,4]triazolo[4,3-a][1,4]diazepin-6-yl)acetic acid
      1.1.3. (S)-2-(4-(4-chlorophenyl)-2,3,9-trimethyl-6H-thieno[3,2-f][1,2,4]triazolo[4,3-a][1,4]diazepin-6-yl)-N-(prop-2-yn-1-yl)acetamide
         1.1.3.1 ^{1}H NMR spectrum
         1.1.3.2 LC-MS spectrum
   1.2. Nucleic acid sequences
      1.2.1 Table 1: Antisense oligonucleotide sequences used
      1.2.2 Table 2: Molecular weights for oligonucleotide conjugates prepared
   1.3. Nucleic Acid Chemistry Functionalisation, Purification, and Characterisation
      1.3.1. Azidoacetic acid-NHS ester functionalisation
      1.3.2. JQ1-functionalisation with copper click chemistry
      1.3.3. Oligonucleotide MS characterisation
   1.4. Biological assays
      1.4.1. Cell Culture
      1.4.2. Transfection
      1.4.3. Gymnosis
      1.4.4. Electroporation
      1.4.5. Luciferase Assay
      1.4.6. BCA Assay
      1.4.7. Competition Assay
      1.4.8. CellTiter-Glo
      1.4.9. RT-qPCR
         1.4.9.1. Table 3: qPCR primers sequences
      1.4.10. Western Blotting
      1.4.11. Immunocytochemistry
      1.4.12. Imaging and image processing

2. **Supplementary Figures**……………………………………………………………………………………….10
   2.1. Figure S1: Reaction and characterisation for azide modification of the SSO
   2.2. Figure S2: Reaction and characterisation for JQ1 modification of the SSO
   2.3. Figure S3: Luminescence values for SSO and azido-SSO activity, transfected with lipofectamine 2000, at 24 hours at concentrations indicated. In all cases, luciferase activity was measured and normalised to untreated cells.
   2.4. Figure S4: Cell protein production for indicated SSO and JQ1-SSO treatment over 24h lipofectamine 2000 transfection quantified by BCA.
   2.5. Figure S5: Viability of the HEK293T upon SSO and JQ1-SSO treatment evaluated by Cell-Titer Glo. Luminescence values for SSO and azido-SSO activity for gymnotic uptake at 96 hours at concentrations indicated. In all cases, luciferase activity was measured and normalised to untreated cells.

2.6. Figure S6: Viability of the HEK293T upon SSO and JQ1-SSO treatment evaluated by Cell-Titer Glo.
2.7. Figure S7: Competition assay between JQ1-SSO conjugate and excess small molecule, (+)-JQ1.
2.8. Figure S8: Reaction and characterisation for azide modification of the MALAT1 gapmer ASO.
2.9. Figure S9: Reaction and characterisation for JQ1 modification of the MALAT1 gapmer ASO.
2.10. Figure S10: RT-qPCR data for MALAT1 knockdown upon lipofectamine transfection of unconjugated-MALAT1 gapmer and azido-MALAT1 gapmer in HEK293T cells for 24 hours at concentrations indicated.
2.11. Figure S11: RT-qPCR data for MALAT1 knockdown upon gymnosis of unconjugated-MALAT1 gapmer and azido-MALAT1 gapmer in HEK293T cells for 96 hours at concentrations indicated.
2.12. Figure S12: Competition assay between JQ1-MALAT1 gapmer and excess small molecule, (+)-JQ1.
2.13. Figure S13: Viability of the HEK293T upon MALAT1 gapmer and JQ1-MALAT1 gapmer treatment evaluated by Cell-Titer Glo.
2.14. Figure S14: RT-qPCR data for MALAT1 knockdown upon lipofectamine transfection of unconjugated-MALAT1 gapmer and (+)-JQ1-MALAT1 gapmer in A549 cells for 24 hours at concentrations indicated.
2.15. Figure S15: Representative immunocytochemistry of HEK293 cells transfected with L2000 only, unconjugated and (+)-JQ1-modified MALAT1 gapmer for 24 hours using antibodies against the PS modifications (green) and α/β-tubulin (red).
2.16. Figure S16: Reaction and characterisation for azide modification of the Oblimersen (G3139) ASO.
2.17. Figure S17: Reaction and characterisation for JQ1 modification of the Oblimersen (G3139) ASO.
2.18. Figure S18: Reaction and characterisation for JQ1 modification of the PS NTC-ASO.
2.19. Figure S19: RT-qPCR data of BCL-2 knockdown upon unconjugated-G3139, azido-G3139, and (+)-JQ1-NTC-ASO lipofectamine transfection in HEK293Ts for 24 hours at concentrations indicated.
2.20. Figure S20: Uncropped western blot of BCL-2 levels upon treatment with G3139 and (+)-JQ1-G3139 upon transfection with lipofectamine at 24 hours.
2.21. Figure S21: RT-qPCR data of BCL-2 knockdown upon unconjugated-G3139, azido-G3139, and (+)-JQ1-NTC-ASO gymnosis in HEK293Ts for 96 hours at concentrations indicated
2.22. Figure S22: Viability of the HEK293T upon G3139 ASO and JQ1-G3139 ASO treatment evaluated by Cell-Titer Glo.
2.23. Figure S23: Reaction and characterisation for azide modification of the Oblimersen (G3139)-Cy3 ASO.
2.24. Figure S24: Reaction and characterisation for JQ1 modification of the Azido-G3139-Cy3.
2.25. Figure S25: RT-qPCR data of BCL-2 knockdown upon G3139-Cy3 and (+)-JQ1-G3139-Cy3 lipofectamine transfection in HEK293Ts for 24 hours at concentrations indicated.
2.26. Figure S26: Western blot for verification of successful cytoplasmic (GAPDH) and nuclear (MENIN) fractionation upon G3139-Cy3 and (+)-JQ1-G3139-Cy3 lipofectamine transfection in HEK293Ts for 24 hours at concentrations indicated.
2.27. Figure S27: Raw Cy3 fluorescence values of cytoplasmic and nuclear fractions upon G3139-Cy3 and (+)-JQ1-G3139-Cy3 lipofectamine transfection in HEK293Ts for 6, 12, and 24 hours at concentrations indicated.
2.28. Figure S28: Representative immunocytochemistry of HEK293 cells transfected with L2000 only, unconjugated and (+)-JQ1-modified G3139 for 24 hours using antibodies against the PS modifications (green) and α/β-tubulin (red).
2.29. Figure S29: RT-qPCR data of BCL-2 knockdown upon unconjugated-G3139, azido-G3139, and (+)-JQ1-NTC-ASO electroporation in THP-1s for 48 hours at concentrations indicated.
2.30. Figure S30: Uncropped western blot of BCL-2 levels in THP-1s upon treatment with G3139 and (+)-JQ1-G3139, two rounds of electroporation at 96 hours at concentrations indicated. Normalised to GAPDH expression levels.
2.31. Figure S31: Uncropped western blot of BCL-2 levels in THP-1s upon treatment with G3139 and (+)-JQ1-G3139, two rounds of electroporation at 96 hours at concentrations indicated. Normalised to GAPDH expression levels (biological replicate).

3. **References**……………………………………………………………………………………………**29**

1.1 Materials and Methods

1.1.1 Organic synthesis

General information

Reagents were purchased from commercial sources (Merck, BroadPharm and Sigma-Aldrich) and used without further purification. Dry solvents were taken from a solvent drying system (MBraun MB-SPS-5-Bench Top) under nitrogen atmosphere (H_2O content < 20 ppm as determined by Karl Fischer titration). Eluent mixtures are reported in %vol and volume:volume. Column chromatography was carried out using an automated Biotage Selekt One purification machine with Biotage Sfär Silica or C18 Bio Duo flash chromatography cartridges. HPLC grade solvents were used for purifications, extractions, and workups. TLC was carried out on Merck silica gel 60 F254 Al plates visualized under UV light (254 nm) or by staining with permanganate.

NMR spectroscopy measurements were recorded using a Bruker AVII400 or AVIII600 instrument and peaks were referenced to the residual solvent peak. Mass Spectrometry (MS) measurements for small molecules synthesised was performed on an ACQUITY I-Class PLUS UPLC System (Waters, Milford, MA, USA) coupled to an ACQUITY RDa mass spectrometer (Waters, Milford, MA, USA) equipped with an ESI probe, in positive ion mode. An ACE Equivalence 3 C18 column (50 x 2.1 mm, 3 µm; Avantor, Radnor, PA, USA) at 40C, was used with mobile phase A: water + 0.1 % formic acid, and mobile phase B: acetonitrile + 0.1% formic acid. The linear gradient used was: 0 minutes, 5% B; 0.5 minutes, 5% B; 4.0 minutes 95% B; 4.25 minutes, 95% B; 4.26 minutes, 5% B; and 5 minutes, 5% B. The flow rate was 0.4 mL/minute and the total analysed time was 5 minutes.

1.1.2 **(S)-2-(4-(4-chlorophenyl)-2,3,9-trimethyl-6H-thieno[3,2-f][1,2,4]triazolo[4,3-a][1,4]diazepin-6-yl)acetic acid**

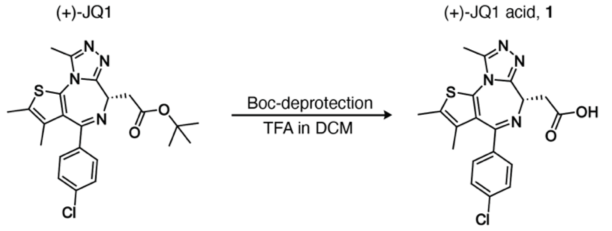

Following a literature procedure[1], to a stirred solution of (+)-JQ1 (90.0 mg, 0.2 mmol) in dichloromethane (4.0 mL) was added trifluoroacetic acid (0.5 mL). The reaction mixture was stirred at room temperature for 2 hours. The reaction mixture was concentrated under vacuum, and used without purification.

1.1.3 **(S)-2-(4-(4-chlorophenyl)-2,3,9-trimethyl-6H-thieno[3,2-f][1,2,4]triazolo[4,3-a][1,4]diazepin-6-yl)-N-(prop-2-yn-1-yl)acetamide**

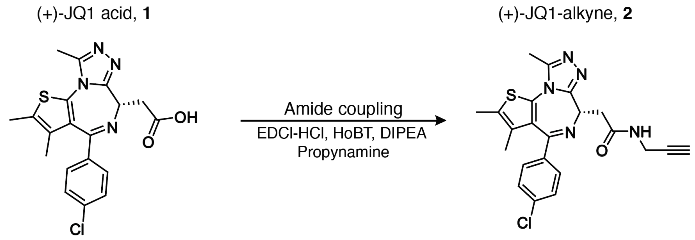

Following a patented procedure[2], to a solution of (+)-JQ1 acid, **1** (160 mg, 0.4 mmol), prop-2-yn-1-amine (26.4 mg; 0.5 mmol), EDCl-HCl (84.2 mg; 0.44 mmol), and HoBT (74.4 mmol; 0.44 mmol) in DMF (2 ml) was added DIPEA (181 mg; 1.4 mmol). The reaction mixture was stirred at room temperature overnight. The reaction mixture was subsequently, diluted with water and extracted with ethyl acetate. The organic layer was washed with brine, dried over Na_2SO_4, and concentrated. The resulting residue was purified by normal phase column chromatography with (0 to 10% MeOH : DCM) to give JQ1-alkyne, **2** (159 mg; 87.8 %) as an off-white oil. ^{1}H NMR (600 MHz, $CDCl_3$) δ 7.42 (d, J = 8.1 Hz, 2H), 7.32 (d, J = 8.6 Hz, 2H), 6.93 (br s, 1H), 4.63 (dd, J = 6.5 Hz, 6.5 Hz, 1H), 4.18 (ddd, J = 17.5 Hz, 5.6 Hz, 2.4 Hz,

1H), 3.97 (ddd, J = 17.5 Hz, 4.5 Hz, 2.3 Hz, 1H), 3.33 (dd, J =14.0 Hz, 6.5 Hz, 1H), 2.60 (s, 3H), 2.33 (s, 3H), 2.14 (t, J = 2.5 Hz, 1H), 1.60 (s, 3H). MS (ESI, m/z): MS (ESI+) found m/z 438.1141 (M+H)+.

1.1.3.1 ^{1}H NMR spectrum

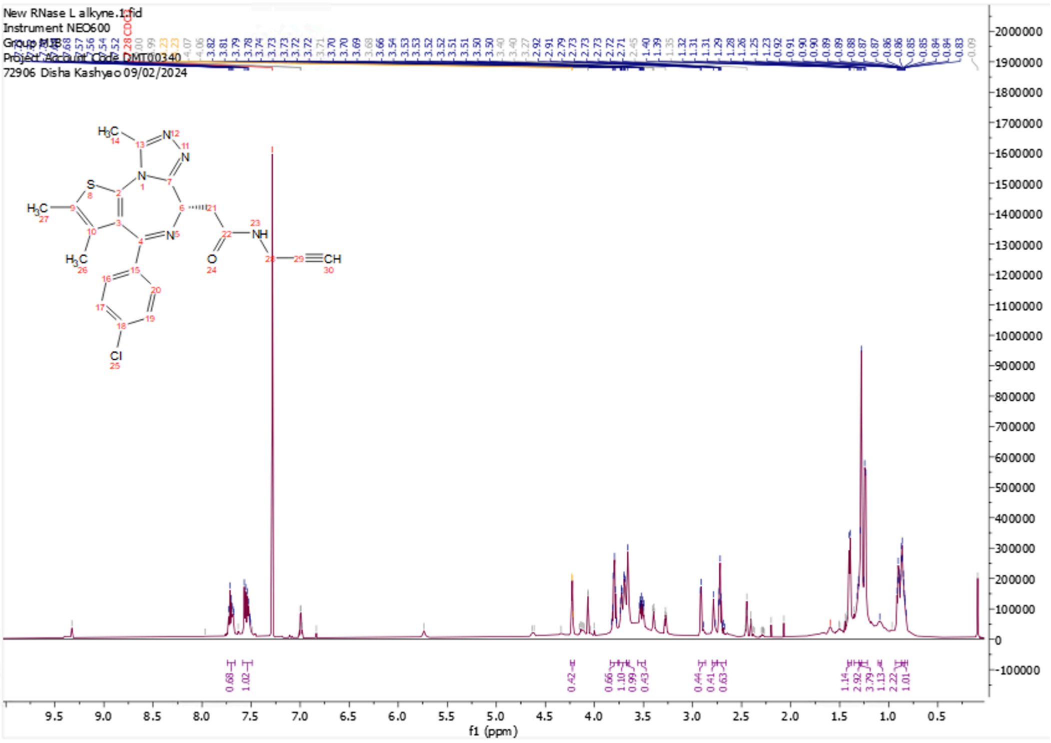

1.1.3.2 LC-MS spectrum

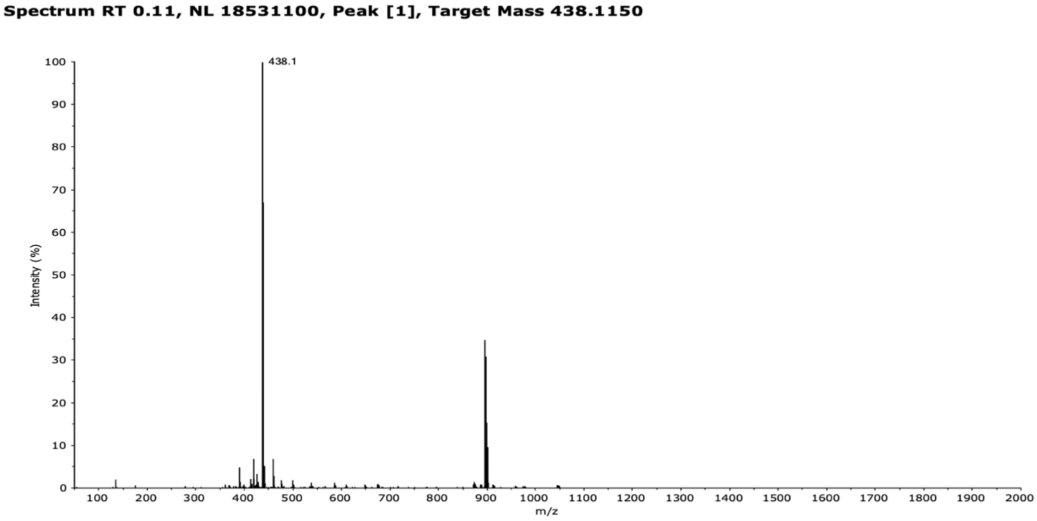

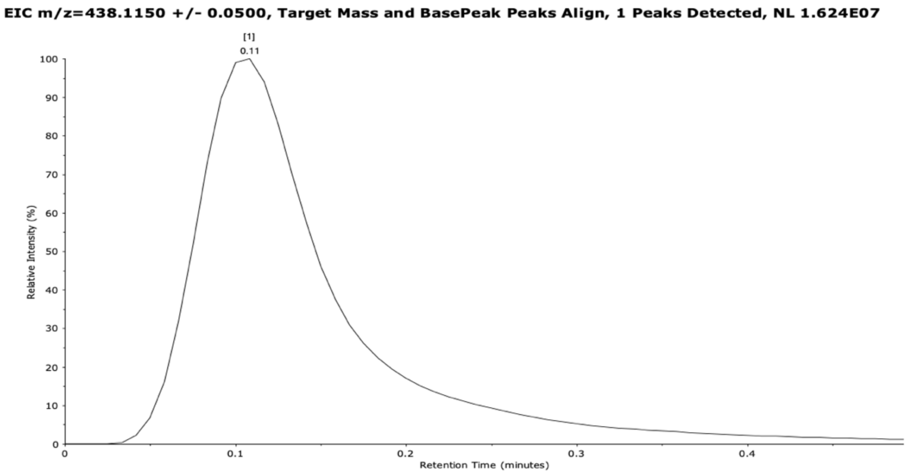

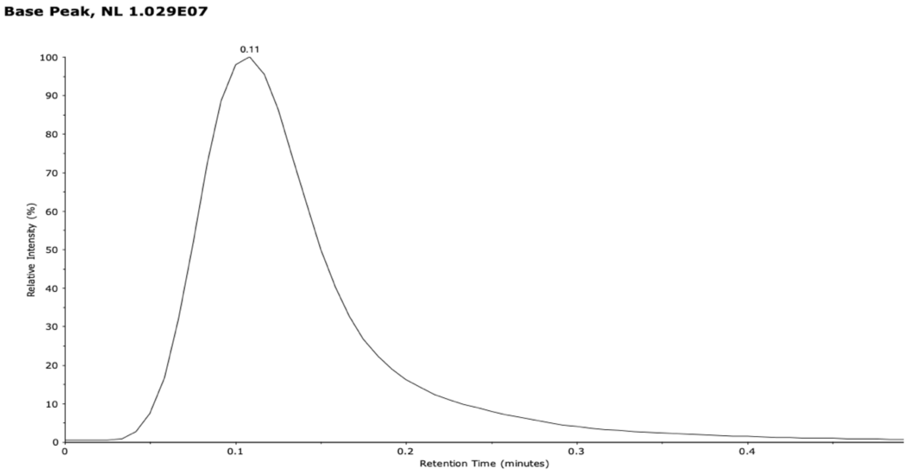

1.2 Nucleic acid sequences

All unmodified oligonucleotides were purchased from IDT as desalted and lyophilised products – resuspended in 10 mM Tris pH 8. Amine-containing oligonucleotides were purchased from IDT, HPLC-purified in lyophilised form and dissolved in 10 mM potassium phosphate buffer, pH 8.

Table 1: Antisense oligonucleotide sequences used for all transfection experiments with 5′-terminal amine or azide modifiers for functionalisation chemistry

* denotes phosphorothioate linkages

_ denotes 2′-Methoxy-ethyl sugars

m denotes 2′-O-Methyl sugars

Name	Sequence (5'-3')
SSO	mC*mC*mU*mC*mU*mU*mA*mC*mC*mU*mC*mA*mG*mU*mU*mA*mC*mA
MALAT1 gapmer	<u>G*G*C*A*T</u>*A*T*G*C*A*G*A*T*A*A*<u>T*G*T*T*C</u>
Oblimersen, G3139	T*C*T*C*C*C*A*G*C*G*T*G*C*G*C*C*A*T
NTC-ASO	T*C*G*T*G*G*G*T*A*G*G*T*C*C*G*C*A*C*T*A

Table 2: Molecular weights for oligonucleotide conjugates prepared

Name	Expected mass	Mass after deconvolution
Azide-SSO	6360.0	**6359.8**
JQ1-SSO	6799.0	**6797.8**
Azide-MALAT1 gapmer	7493.0	**7491.6**
JQ1-MALAT1 gapmer	7930.0	**7928.6**
Azide-G3139	5947.0	**5945.5**
JQ1-G3139	6385.0	**6383.6**
JQ1-NTC-ASO	7239.0	**7239.5**
Azide-G3139-Cy3	6588.0	**6590.0**
JQ1-G3139-Cy3	7034.0	**7030.0**

1.3 Nucleic acid chemistry functionalisation, purification, and characterisation

Azidoacetic acid-NHS ester functionalisation

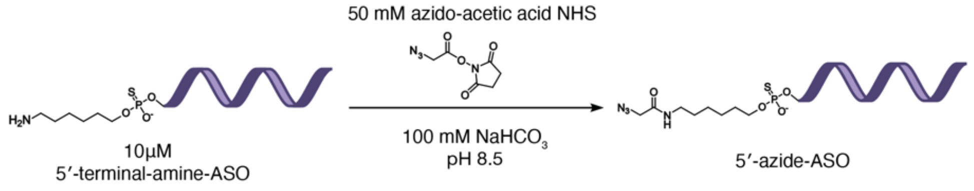

To a 0.5 mL Eppendorf DNA LoBind tube was added 1 µL of the DNA (100 µM stock concentration), 1 µL of $NaHCO_3$ pH 8.5 (1 M), 2 µL of H_2O, 5 µL of a 50 mM solution of azido-acetic acid NHS in dry DMF. The reaction was vortexed, spun down in a tabletop centrifuge and placed in a Thermomixer (Eppendorf) overnight, shaking at 800 rpm at 37°C.

The excess hydrolysed small molecule was removed using Amicon 3K 0.5 mL spin columns, as per the manufacturer's instructions. Briefly, the reaction was made up to a volume of 500 µL with H_2O and spun at 14,700g for 10 minutes. The eluent was removed and the above process was repeated for a total of three times. The column was then inverted, inserted into a fresh collection tube and spun at 14,700g for 1 minute. The eluent obtained was then purified by HPLC on an Agilent Polaris C18 column (150 x 4.6 mm), column heated to 50°C using a gradient of 3-30% CH_3CN (indicated in **SI** figures) over 20 minutes, flow rate of 1.5 mL/min, with 10 mM triethylammonium bicarbonate (TEAB) pH 8.5 as an ion-pairing buffer throughout.

JQ1-functionalisation with copper click chemistry

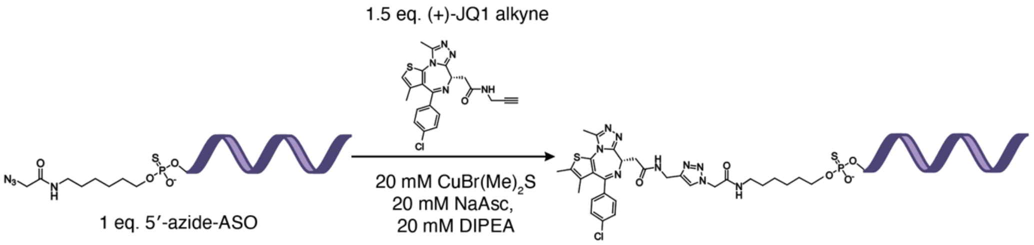

To a 0.5 mL Eppendorf DNA LoBind tube was added, listed in order of addition, 1 µL of the PS-modified DNA (1 mM stock concentration), 1.5 µL of 1 mM JQ1-alkyne, molecule **2**, 1 uL of 200 mM DIPEA, 5.5 µL of H_2O, 1 µL of 200

mM sodium ascorbate and finally, 1 µL of 200 mM copper (I) bromide-dimethyl sulphide. The reaction was vortexed, spun down in a tabletop centrifuge and placed in a Thermomixer (Eppendorf) overnight, shaking at 800 rpm at room temperature.

The reaction was quenched with 250 µL of 0.5 M EDTA pH 8, made up to 500 µL with H_2O and first run through a Amicon 3K 0.5 mL column/tube as described above to chelate excess copper and remove it along with the unreacted small molecule. The final eluent collected after inversion was then purified by HPLC on an Agilent Polaris C18 column (150 x 4.6 mm), column heated to 50°C using a gradient of 3-30% or 3-50% CH_3CN (indicated in **SI** figures) over 20 minutes, flow rate of 1.5 mL/min, with 10 mM triethylammonium bicarbonate (TEAB) pH 8.5 as an ion-pairing buffer throughout.

Oligonucleotide MS characterisation

Oligonucleotide Mass Spectra were recorded on a Waters Xevo G2 QTOF ESI-UPLC-MS system. A gradient of MeOH in Et_3N and hexafluoroisopropanol (HFIP) was used (buffer A, 8.6 mM Et_3N, 200 mM HFIP in 5% MeOH/H_2O (v/v); buffer B, 20% buffer A in MeOH). Data was then deconvoluted using MassLynx software v4.1 and recorded in **table 2**.

1.4 Biological assays

Cell culture

HeLa pLuc/705, HEK293T, A549 cells were both cultured in Dulbecco's Modified Eagle Medium (DMEM) supplemented with 1X GlutaMAX (Gibco) and 10% (v/v) Foetal Bovine Serum (FBS) (Gibco) and) at 37 °C in a humidified incubator with 5% CO_2. HEK293 cells were cultured in DMEM supplemented with 1X GlutaMAX (Gibco), 10% FBS (Gibco), 1% penicillin-streptomycin (Gibco), 1% HEPES (Gibco) and 1% sodium pyruvate (Gibco), and maintained at 37°C in a humidified incubator with 5% CO_2. THP-1 cells were cultured in Roswell Park Memorial Institute 1640 Medium supplemented with 1X GlutaMAX (Gibco) and 10% (v/v) FBS (Gibco) at 37 °C in a humidified incubator with 5% CO_2.

Transfection

For transfection with lipofectamine 2000, HeLa pLuc/705 cells were seeded at a density of 1×10^4 cells/well in 100 µL of culture media in 96-well plates (Corning) for 16 hours before transfection to reach 70-80% cell confluency. For HEK293T \ A549 cells, 2.5×10^5 \ 2.2×10^5 cells were plated in 1 mL of culture media in 24-well plates (Corning) 16 hours before transfection to reach 70-80% cell confluency. Prior to transfection, the culture media was replaced with OptiMEM, 100 µL for the 96-well plates and 1 mL for the 24-well plates. When using HEK293 cells for immunocytochemistry, cells were plated at a density of 5×10^4 cells/well in an 8-well chamber slide (Nunc, Thermo Fischer Scientific) in 400 µL of culture media 24 hours before transfection to reach 70-80% cell confluency. Prior to transfection, culture media was replaced with 180µl fresh complete media.

For the HeLa pLuc/705 cells, 5 µL of Lipofectamine 2000 (Invitrogen) was diluted in 495 µL OptiMEM (Gibco) and incubated at room temperature for 5 minutes before mixing with 4 nmol of oligonucleotide dissolved in 500 µL of OptiMEM[3]. The resulting mixture was incubated at room temperature for 20 minutes allowing complexation to occur. The complexes were then added at the required concentrations to the cells (with total volume at 100 µL per well). The cells were then incubated at 37 °C in a 5% CO_2 incubator. After 6 hours the media was replaced with 100 µL of culture media and the cells were returned to the incubator for a further 18 hours.

For the HEK293T \ A549 cells \ HEK293, 2 µL of Lipofectamine 2000 (Invitrogen) was added to 48 µL OptiMEM (Gibco) and incubated at room temperature for 5 minutes before mixing with 0.5 nmol of oligonucleotide dissolved in 50 µL of OptiMEM. The resulting mixture was incubated at room temperature for 20 minutes allowing complexation to occur. The complexes were then added to the cells at the required concentrations (with total volume of 1 mL per well). The cells were then incubated at 37 °C in a 5% CO_2 incubator. After 6 hours the media was replaced with 1 mL of culture media and the cells were returned to the incubator for another 18 hours.

For HEK293, briefly, as above OptiMEM-Lipofectamine 2000 complexes were prepared by mixing the required volume of OptiMEM (Gibco) and the required volume of Lipofectamine 2000 (Invitrogen) and incubated at room temperature for 5 minutes. The required volumes of ASOs to achieve a 200nM or 500nM dose were added to the OptiMEM-

Lipofectamine 2000, and the transfection mix was incubated for 20 minutes at room temperature to allow for ASO-Lipofectamine 2000 complex formation. 40µl of the transfection mix was added to the wells, and the cells were returned to the incubator for 24 hours.

Gymnosis

HEK293T cells were seeded at a density of 5 × 10^4 cells/well in 1 mL of culture media in 24-well plates for 16 hours before transfection to reach 70-80% cell confluency. Prior to transfection, the culture media was replaced with OptiMEM and the ASO was added at the desired concentration. After 6 hours, OptiMEM was replaced with 1 mL of complete culture media and the cells were returned to the incubator for a further 90 hours (total time of 96 hours).

Electroporation

THP-1 cells were resuspended in complete media at a density of 10^8 cells/mL and the ASOs were added directly to the cell suspension at the desired final concentration. Cells were subjected to a 10 ms 330 V electroporation using a rectangle pulse EPI 2500 electroporator (Fischer, Heidelberg), after which they were diluted to 10^6 cells/mL. Cells were either harvested at 48h, or at this point, a second electroporation was performed, and cells were harvested after a further 48h for a 96h knockdown.

Luciferase assay

The culture media was removed from the well and the cells were washed with 200 µL of PBS. 100 µL of GloLysisTM buffer (Promega) was added to each well and the plate was incubated at room temperature on the orbital shaker for 10 minutes to lyse the cells. 50 µL of the cell lysate was added to 50 µL of Bright-GloTM luciferase reagent (Promega) in a white 96-well plate and the luminescence was measured using a CLARIOstar microplate reader (BMG Labtech). The luminescence values were normalised to the values for untreated cells.

BCA protein assay

25 µL of the cell lysate (as prepared above) was then used for protein quantification using a Pierce BCA protein assay according to the manufacturer's instructions, using the GloLysis buffer as a blank standard. Briefly, a working reagent (WR) was prepared by mixing 50 parts of BCA reagent A with 1 part of BCA reagent B. To 25 µL protein lysate of each sample in a 96-well plate, 200 µL WR was added. The plate was covered with foil and incubated for 1 hour at 37°C in a 5% CO_2 incubator. Absorbance values were corrected for the GloLysis buffer and titrated against known protein standards for quantification.

Competition assay

For all competition assays, cells were co-treated with the specified concentrations of (+)-JQ1 dissolved in DMSO and ASOs. Transfections were performed as described previously, and the cells were treated with (+)-JQ1 treatment concurrently with the addition of the lipofectamine-nucleic acid complexes. Luciferase assay was then performed as described above for the SSO and JQ1-SSO conjugate and qPCR was performed as described below for MALAT1 gapmer, JQ1-MALAT1, G3139 and JQ1-G3139.

CellTiter-Glo

Transfection experiments were carried out as outlined above. Cells were subjected to the CellTiter-Glo assay at the indicated time points following the manufacturer's guidelines. Briefly, 100 µL CellTiter-Glo Reagent was added to 100 µl of media containing cells in a white (Thermofischer Nunc MicroWell polystyrene, 236105) 96-well plate. Subsequently, the contents of the wells were mixed on an orbital shaker for 2 minutes to induce cell lysis. The plate was then incubated at room temperature for 10 minutes to stabilize the luminescent signal. The luminescence was measured using a CLARIOstar microplate reader (BMG Labtech). The luminescence values were normalised to the values for untreated cells.

RT-qPCR

Total RNA was extracted and DNase I-treated from pellets with 1 × 10^6 cells using the RNeasy Mini kit (Qiagen). RNA

was reverse-transcribed using SuperScript III (ThermoFisher Scientific) with random hexamer primers, and then quantified using SYBR Green in QuantStudio 3 Real-time PCR machine (qPCR primers listed in Table 3). Gene expression was normalized to mature mRNA levels of the housekeeping gene, GAPDH.

Table 3: qPCR primer sequences, using SYBR for knockdown quantification

S. No.	Name	Sequence (5'-3')
1	GAPDH FWD	TTGGCTACAGCAACAGGGTG
2	GAPDH REV	GGGGAGATTCAGTGTGGTGG
3	MALAT1 FWD	GAAGGAAGGAGCGCTAAC
4	MALAT1 REV	TACCAACCACTCGCTTTCCC
5	BCL2 FWD	CCCTGTGGATGACTGAGTACCTG
6	BCL2 REV	CCAGCCTCCGTTATCCTGG

Western blotting

Salt-soluble proteins were extracted from 1×10^6 cells by incubating cells in a high-salt lysis buffer (20 mM Tris-HCl pH 8.0, 300 mM KCl, 5 mM EDTA, 20% glycerol, 0.5% IGEPAL CA-630, protease inhibitor cocktail). Protein extracts were then run on a NuPAGE 12% BisTris gels (Life Technologies) at 180V for 1 hour and blotted onto a polyvinylidene fluoride membrane (Immobilon) at 100V for 1 hour using a Tris-glycine blotting buffer.[4] The blots were then probed with 1:10,000 dilution of primary antibody for BCL-2 (CST #4223, D55G8) in 5% milk/TBS-tween at 4°C overnight. BCL-2 blots were then probed with secondary antibody (ab216773, IRDye 800CW) for 2 hours at room temperature, followed by imaging on the ChemiDoc MP system.

Immunocytochemistry (ICC)

Nuclear stain was prepared by diluting Hoechst 3342 dye (Invitrogen) in PBS to obtain a 1:2000 dilution. Cell media post-transfection was removed, and 400 µL of nuclear stain was added followed by an incubation for 5 minutes at room temperature on a shaker with protection from light. The nuclear stain was then removed, and the cells were washed with PBS 3 times for 5 minutes each. Next, 4% paraformaldehyde (PFA) solution was prepared by diluting a 16% PFA stock (Thermo Scientific) in PBS. The PBS from the washes was removed, and 400 µL of 4% PFA was added to the cells followed by incubation for 10 minutes at room temperature on a shaker with protection from light. Then the PFA solution was removed, and the cells were washed 3 times with PBS for 5 minutes each. Next, the permeabilization buffer was prepared by diluting Triton-X (Bio-Rad) in PBS to obtain a 0.1% solution. The PBS from the washes was then removed and 400 µL of 0.1% permeabilization buffer was added followed by incubation for 10 minutes at room temperature on a shaker with protection from light. Blocking buffer was then prepared by diluting goat serum (Gibco) in permeabilization buffer to obtain a 5% solution. Thereafter, the permeabilization buffer was removed, 400 µl of 5% blocking buffer was added and incubated for 1 hour at room temperature on a shaker with protection from light. The primary antibody dilutions were prepared next. The primary antibodies PS03 (mouse) (Rockland Immunochemicals) and α/β-tubulin (rabbit) (Cell Signalling Technology) were diluted in blocking buffer to obtain 1:1000 and 1:500 dilutions respectively. The blocking buffer was removed and 400 µL of diluted antibodies was added followed by incubation for 1 hour at room temperature on a shaker with protection from light. Diluted primary antibodies were then removed, and the cells were washed 3 times with PBS for 5 minutes each. Thereafter, the secondary antibodies anti-mouse AF488 (Invitrogen) and anti-rabbit AF467 (Invitrogen) were diluted in blocking buffer to obtain 1:2000 dilutions each. The PBS from the washes was removed and 400 µL of the diluted secondary antibodies was added followed by incubation for 1 hour at room temperature on a shaker with protection from light. The cells were then washed 3 times with PBS for 5 minutes each for the final time. Slides were then mounted in Fluoromount-G mounting medium (Invitrogen).

Imaging and image processing

Imaging was performed on a Zeiss LSM 900 confocal microscope equipped with a 63x objective (immersion oil) (Carl Zeiss). 2-D images and 3-D Z-stack images were acquired on ZEN blue (Zeiss) software 3.7 with the following parameters: bidirectional scanning, 4x averaging, 16 bits per pixel, 10-20 slices with a range of 10-20 µm and interval of 1 µm. Images were processed and analysed using Zeiss ZEN software (Carl Zeiss) and Fiji (NIH). For each treatment, three randomly selected fields of view were used to generate 2-D images and Z-stacks. For each Z-stack

image, maximum intensity projections were generated, and the areas of AF488 within the nuclei and Hoechst 3342 signals were measured. Statistical analysis was performed using the GraphPad Prism 10.4.1 software. Data are presented as mean ± standard error of the mean (SEM). Differences between groups were analysed using One-Way Analysis of Variance (ANOVA) followed by Šídák's multiple comparisons test.

2. Supplementary Figures 1-31

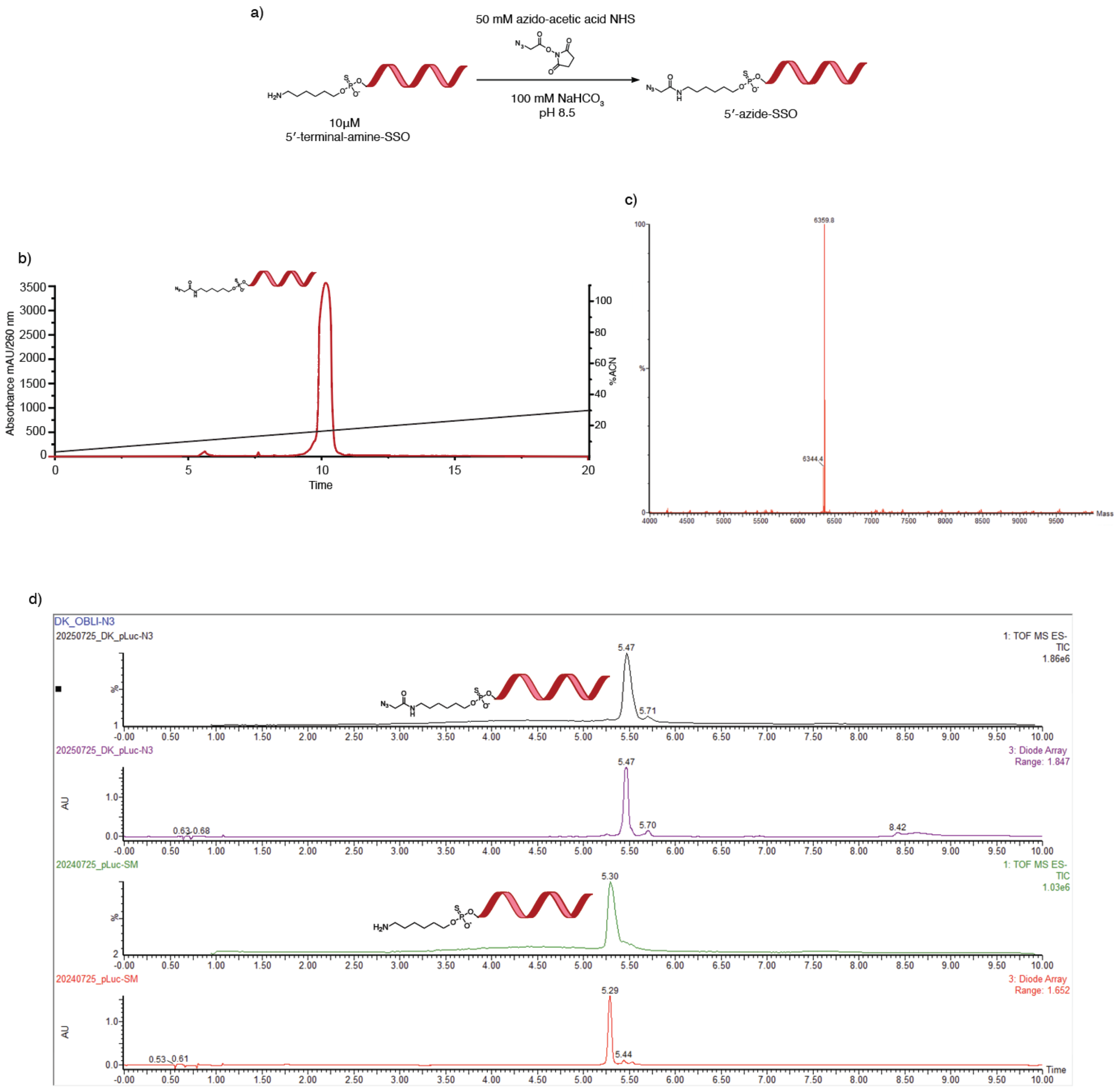

Supplementary Figure 1. Reaction and characterisation for azide modification of the SSO. **a**) Reaction scheme for azide functionalisation of SSO. **b**) HPLC purification for azide-SSO functionalisation. **c**) LC-MS characterisation for HPLC-purified azide-SSO. **d**) Mass spectrum for HPLC-purified azide-SSO

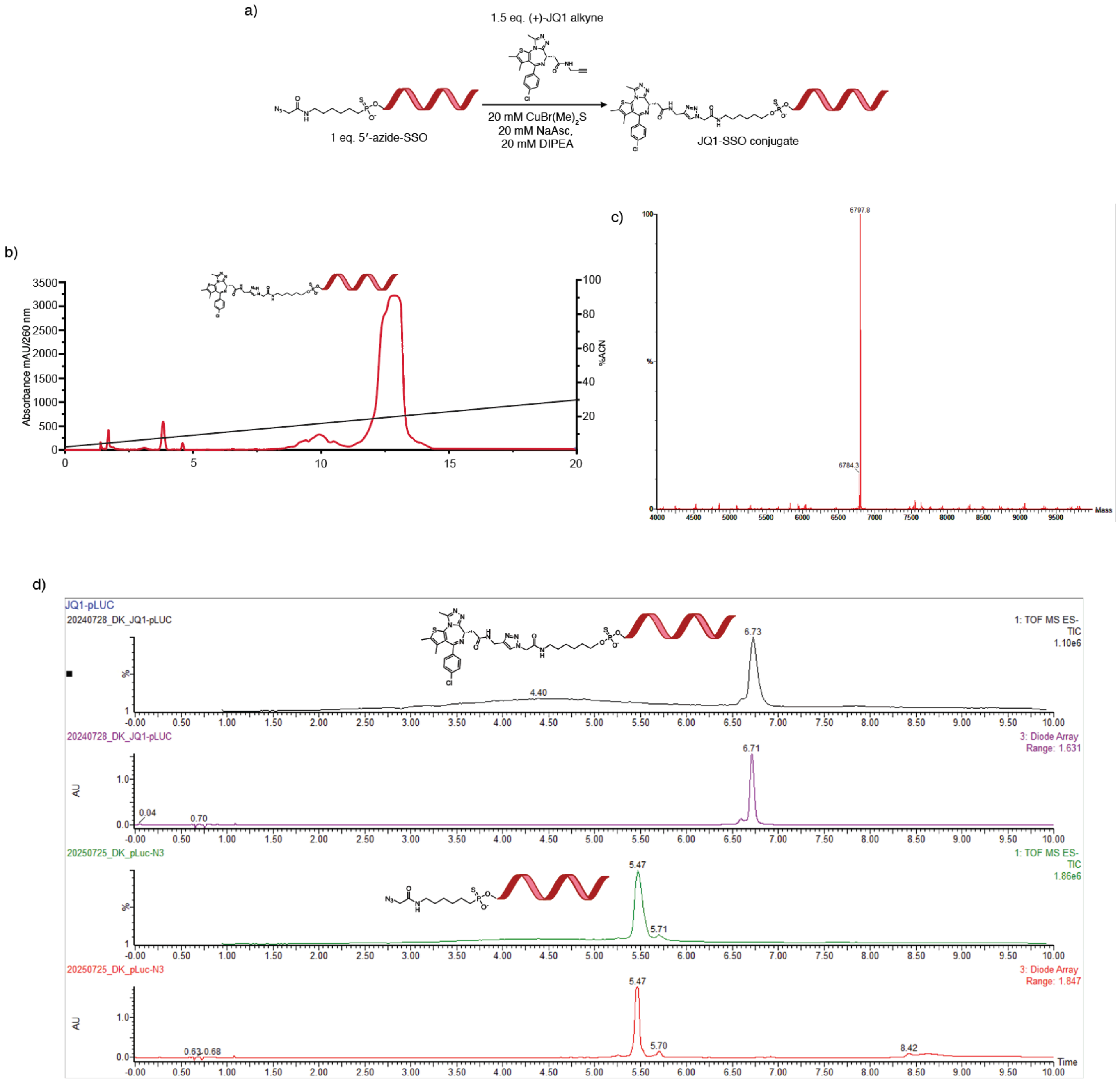

Supplementary Figure 2. Reaction and characterisation for JQ1 modification of the SSO. **a**) Reaction scheme for copper click conjugation of JQ1-alkyne with azide-SSO. **b**) HPLC purification for JQ1-SSO functionalisation. **c**) LC-MS characterisation for HPLC-purified JQ1-SSO. **d**) Mass spectrum for HPLC-purified JQ1-SSO.

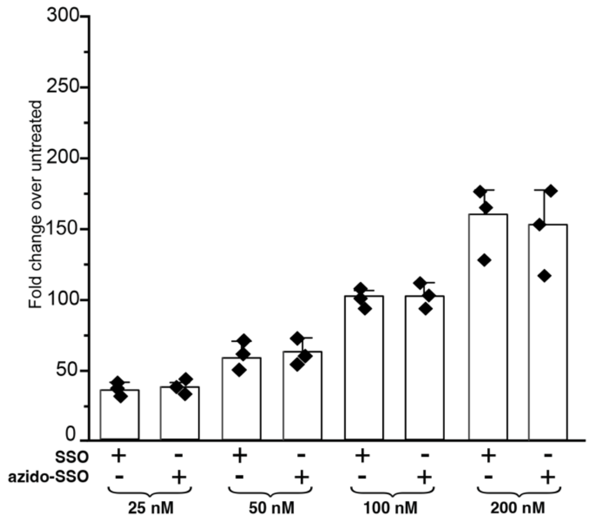

Supplementary Figure 3. Luminescence values for SSO and azido-SSO activity, transfected with lipofectamine 2000, at 24 hours at concentrations indicated. In all cases, luciferase activity was measured and normalised to untreated cells.

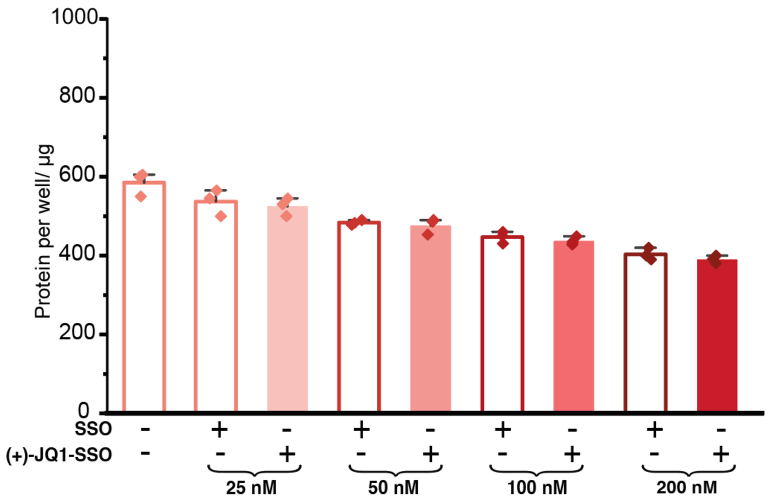

Supplementary Figure 4. Cell protein production for indicated SSO treatment over 24h lipofectamine 2000 transfection quantified by BCA.

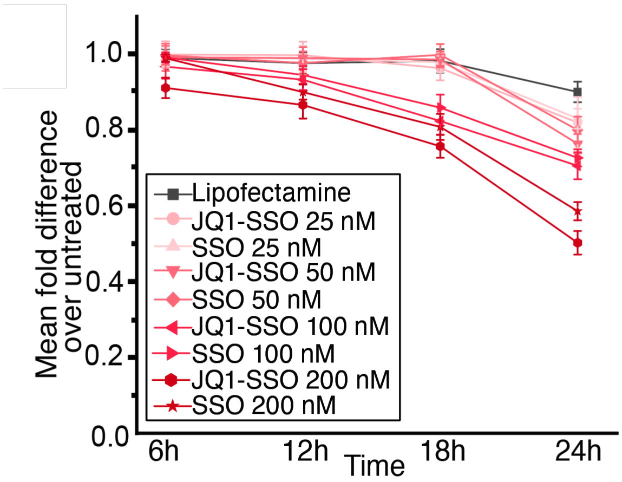

Supplementary Figure 5. Viability of the HEK293T upon SSO and JQ1-SSO treatment evaluated by Cell-Titer Glo.

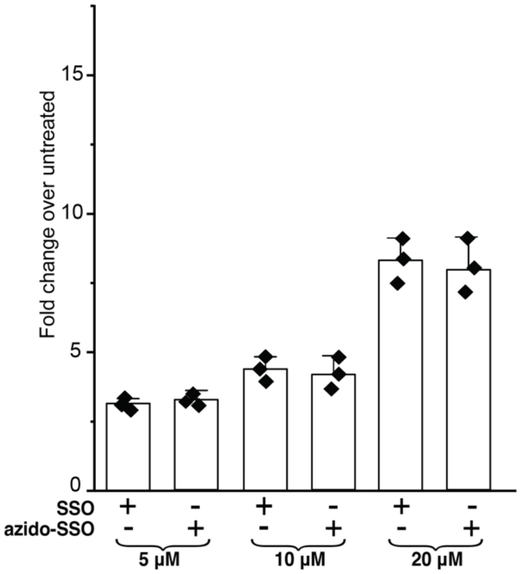

Supplementary Figure 6. Luminescence values for SSO and azido-SSO activity for gymnotic uptake at 96 hours at concentrations indicated. In all cases, luciferase activity was measured and normalised to untreated cells.

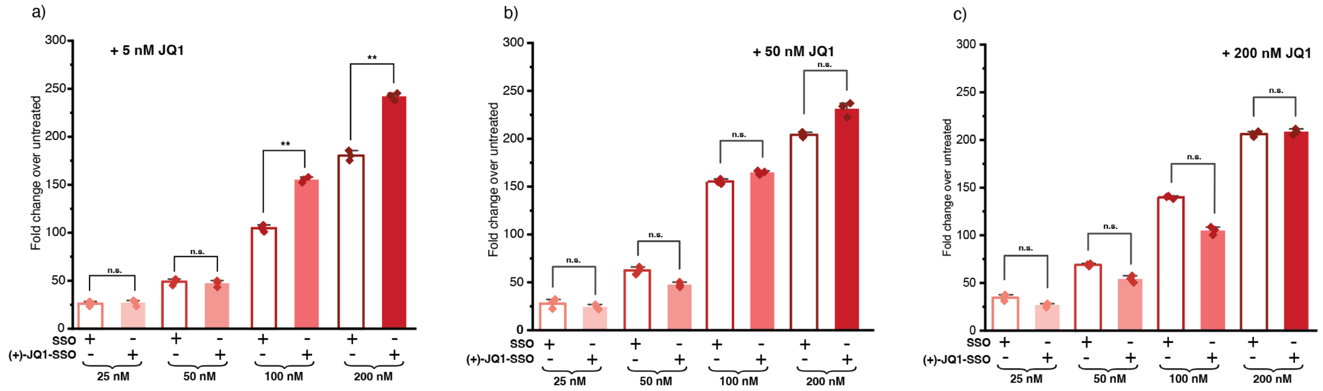

Supplementary Figure 7. Competition assay between JQ1-SSO conjugate and excess small molecule, (+)-JQ1 at **a**) 5nM, **b**) 50 nM, **c**) 200 nM. Error bars represent standard deviation. ** represents p<0.05, *** represents p<0.01, n.s. represents p value not significant.

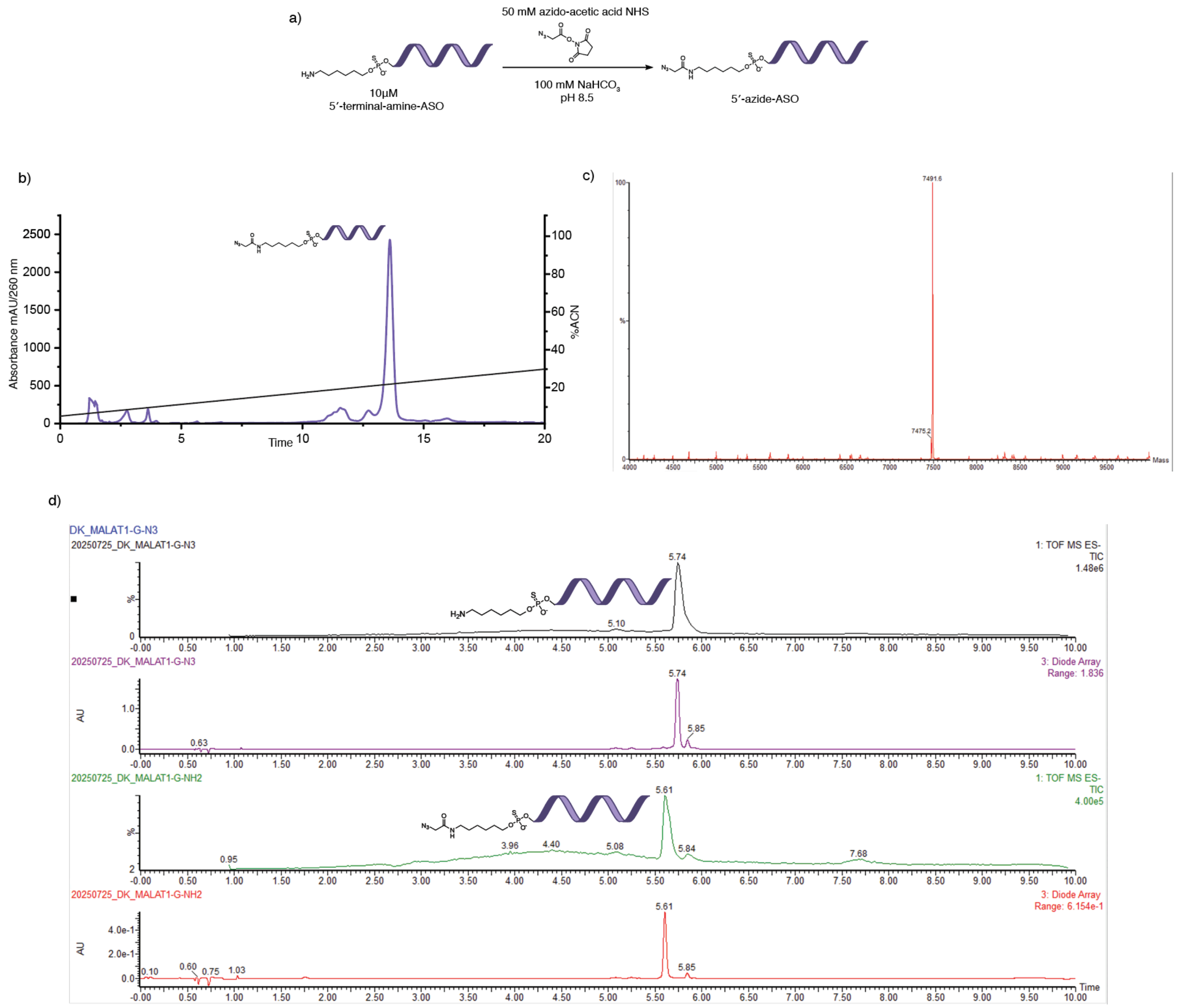

Supplementary Figure 8. Reaction and characterisation for azide modification of the MALAT1 gapmer ASO. **a**) Reaction scheme for azide functionalisation of MALAT1 gapmer. **b**) HPLC purification for azide-MALAT1 gapmer functionalisation. **c**) LC-MS characterisation for HPLC-purified azide-MALAT1 gapmer. **d**) Mass spectrum for HPLC-purified azide-MALAT1 gapmer.

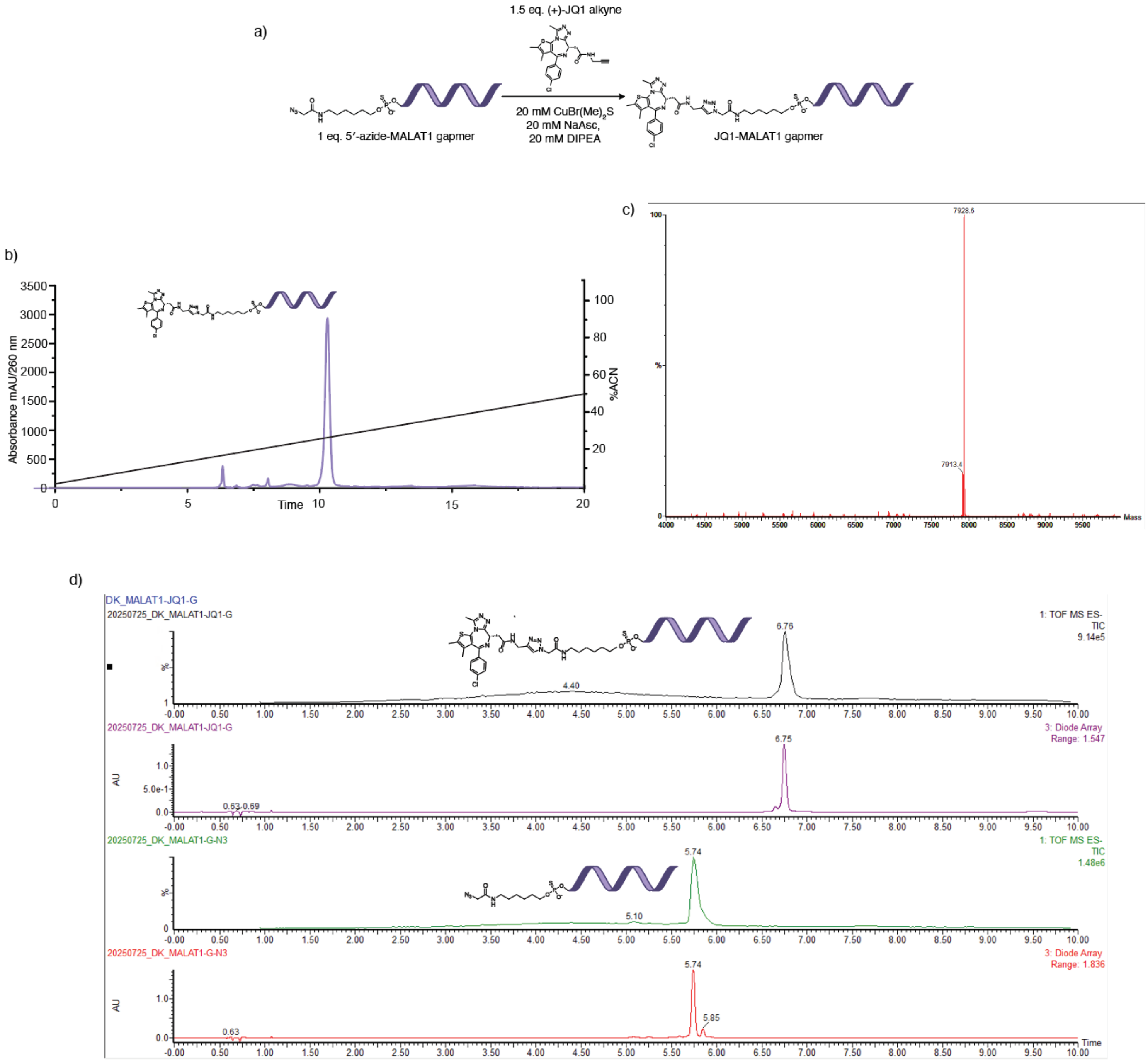

Supplementary Figure 9. Reaction and characterisation for JQ1 modification of the MALAT1 gapmer ASO. **a**) Reaction scheme for copper click conjugation of JQ1-alkyne with azide-MALAT1 gapmer. **b**) HPLC purification for JQ1-MALAT1 gapmer functionalisation. **c**) LC-MS characterisation for JQ1-MALAT1 gapmer. **d**) Mass spectrum for HPLC-purified JQ1-MALAT1 gapmer.

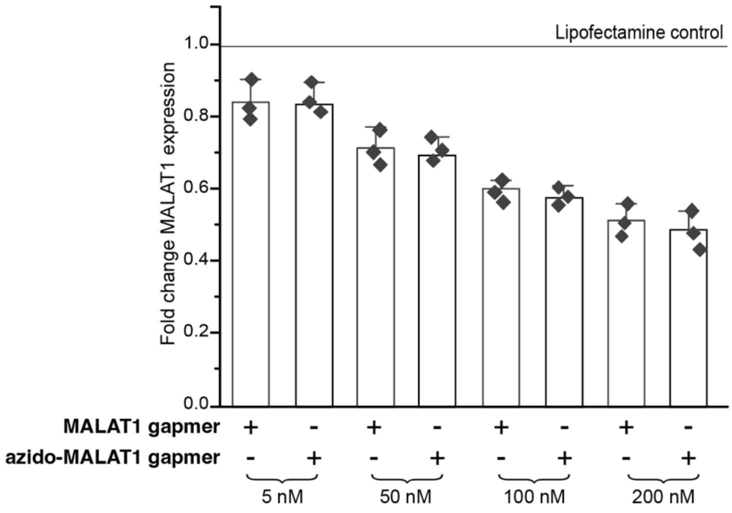

Supplementary Figure 10. RT-qPCR data for MALAT1 knockdown upon lipofectamine transfection of unconjugated-MALAT1 gapmer and azido-MALAT1 gapmer in HEK293T cells for 24 hours at concentrations indicated.

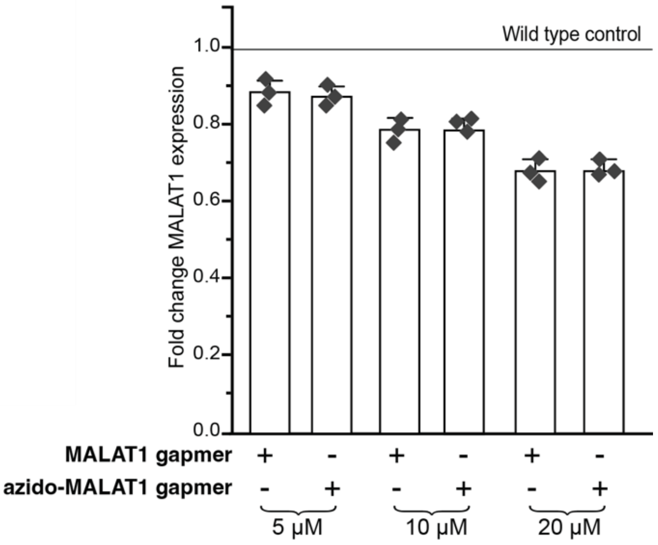

Supplementary Figure 11. RT-qPCR data for MALAT1 knockdown upon gymnosis of unconjugated-MALAT1 gapmer and azido-MALAT1 gapmer in HEK293T cells for 96 hours at concentrations indicated.

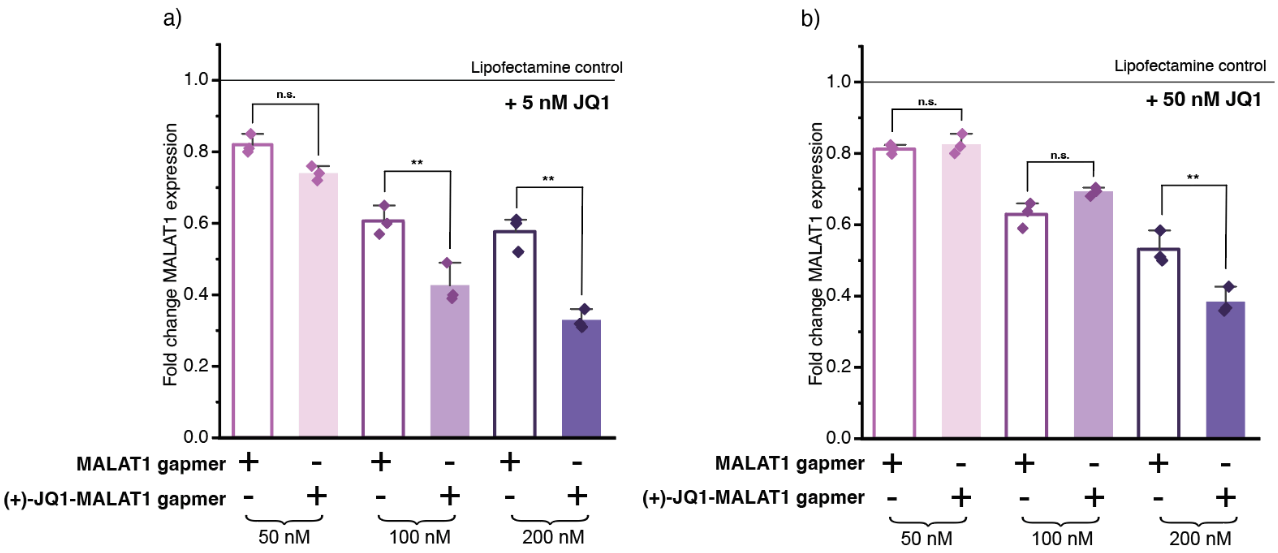

Supplementary Figure 12. Competition assay between JQ1-MALAT1 gapmer and excess small molecule, (+)-JQ1 at **a**) 5nM and **b**) 50 nM. Error bars represent standard deviation. ** represents p<0.05, *** represents p<0.01, n.s. represents p value not significant.

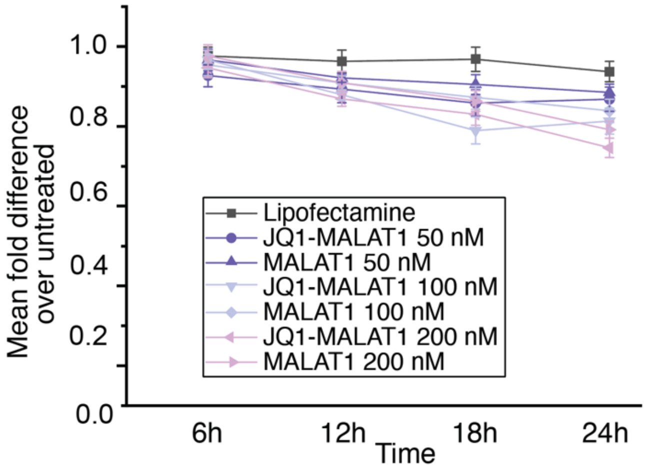

Supplementary Figure 13. Viability of the HEK293T upon MALAT1 gapmer and JQ1-MALAT1 gapmer treatment evaluated by Cell-Titer Glo.

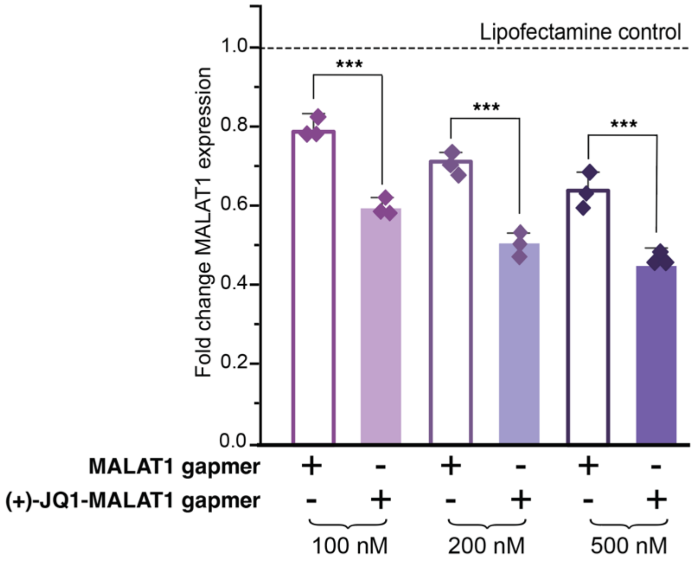

Supplementary Figure 14. RT-qPCR data for MALAT1 knockdown upon lipofectamine transfection of unconjugated-MALAT1 gapmer and (+)-JQ1-MALAT1 gapmer in A549 cells for 24 hours at concentrations indicated.

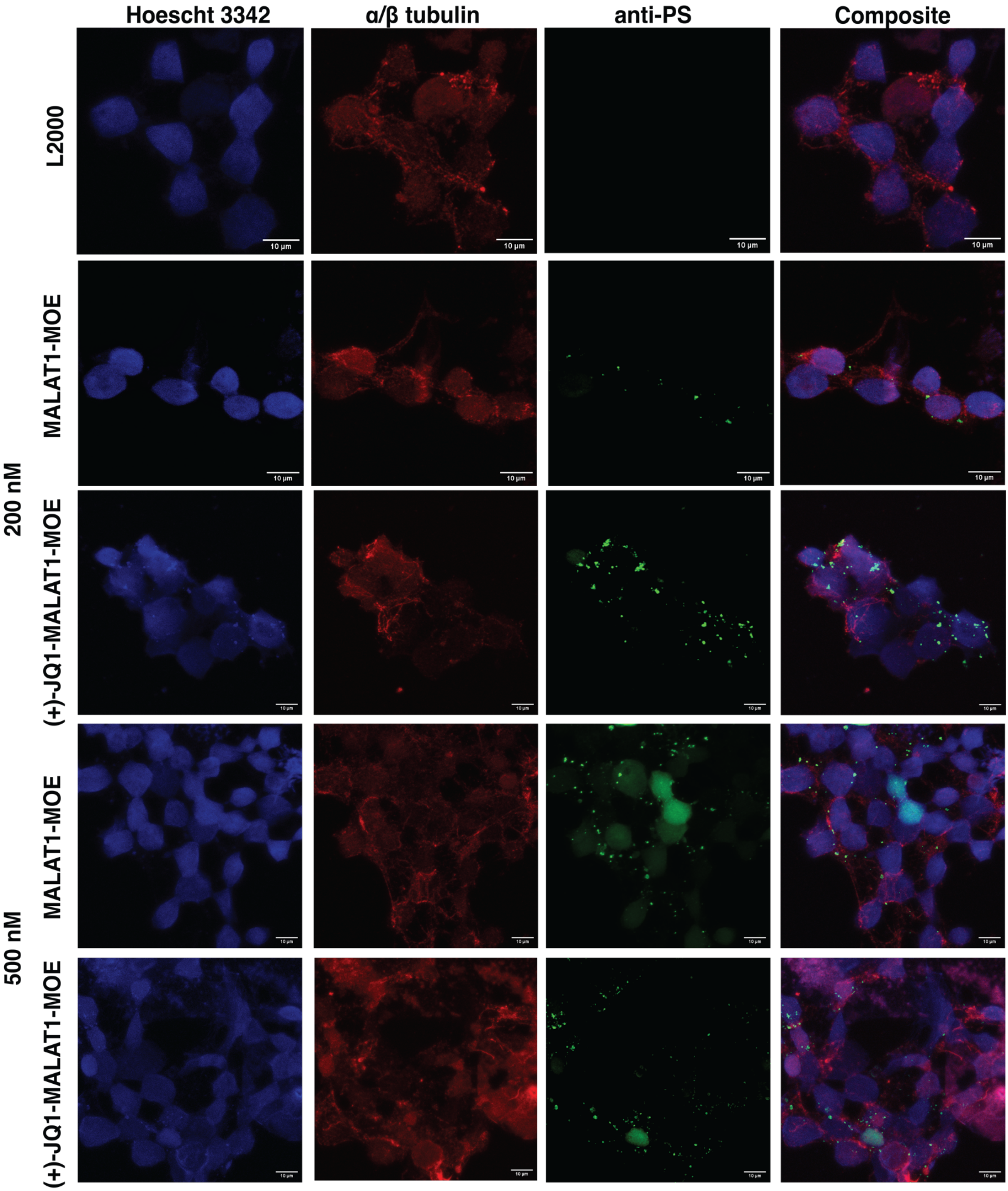

Supplementary Figure 15. Representative immunocytochemistry of HEK293 cells transfected with L2000 only, unconjugated and (+)-JQ1-modified MALAT1 gapmers at concentrations indicated for 24 hours using antibodies against the PS modifications (green) and α/β–tubulin (red). Images are maximum intensity projections generated from Z-stacks; magnification 63x, scale bars as indicated.

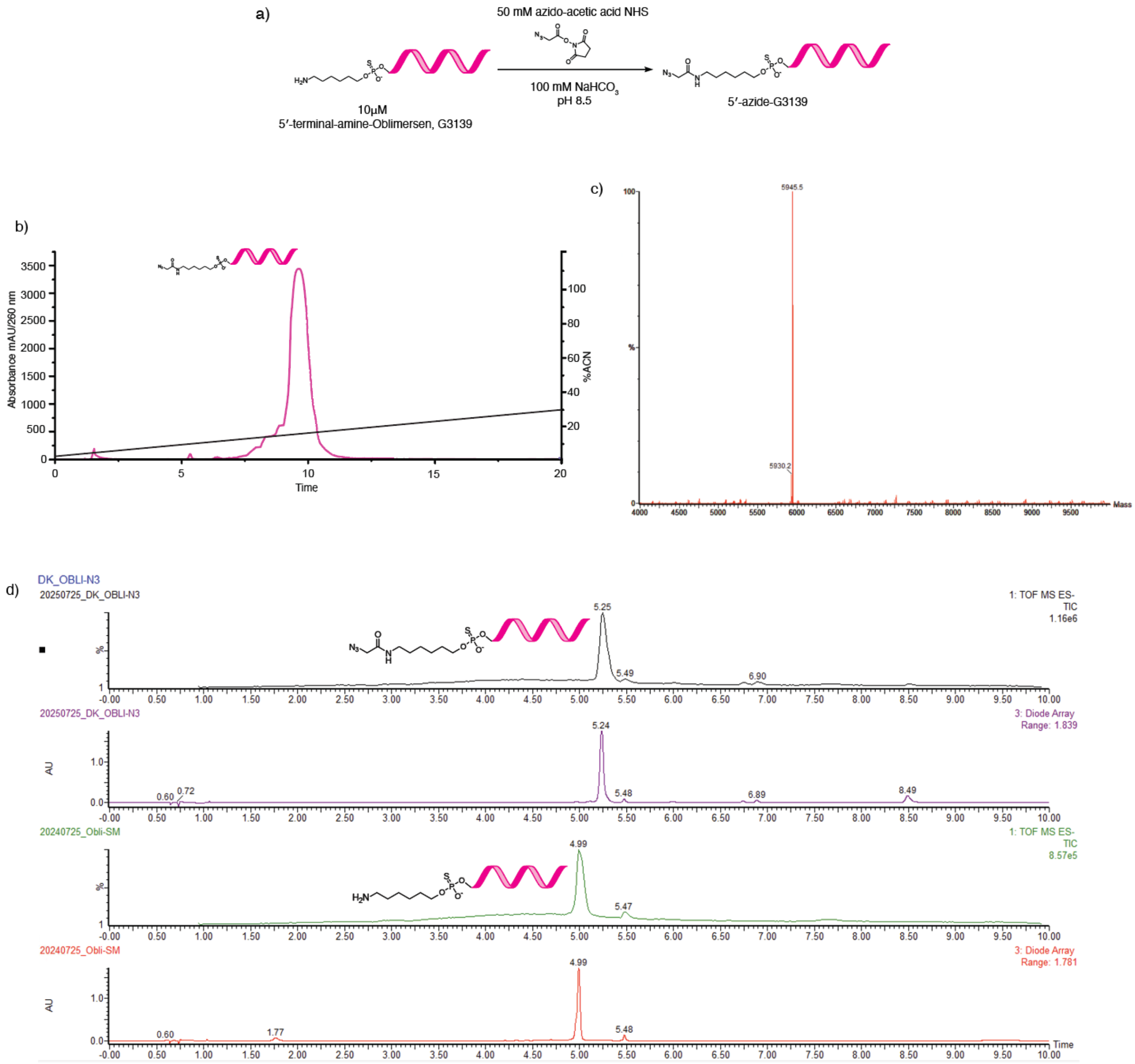

Supplementary Figure 16. Reaction and characterisation for azide modification of the Oblimersen (G3139) ASO. **a**) Reaction scheme for azide functionalisation of Oblimsersen, G3139. **b**) HPLC purification for azide-G3139 functionalisation. **c**) LC-MS characterisation for HPLC-purified azide-G3139. **d**) Mass spectrum for HPLC-purified azide-G3139.

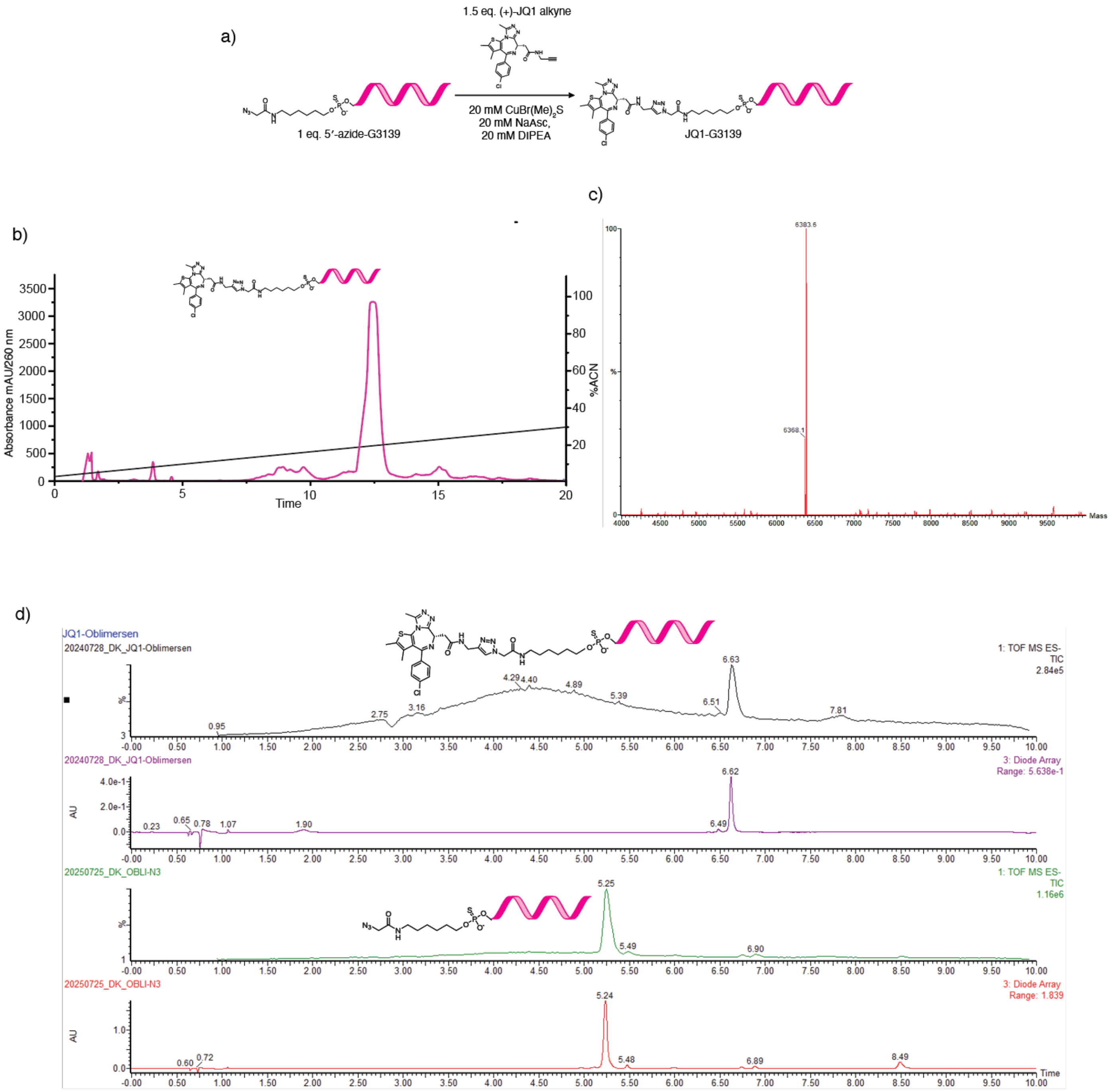

Supplementary Figure 17. Reaction and characterisation for JQ1 modification of the Oblimersen (G3139) ASO. **a**) Reaction scheme for copper click conjugation of JQ1-alkyne with azide-G3139. **b**) HPLC purification for JQ1-G3139 functionalisation. **c**) LC-MS characterisation for HPLC-purified JQ1-G3139. **d**) Mass spectrum for HPLC-purified JQ1-G3139.

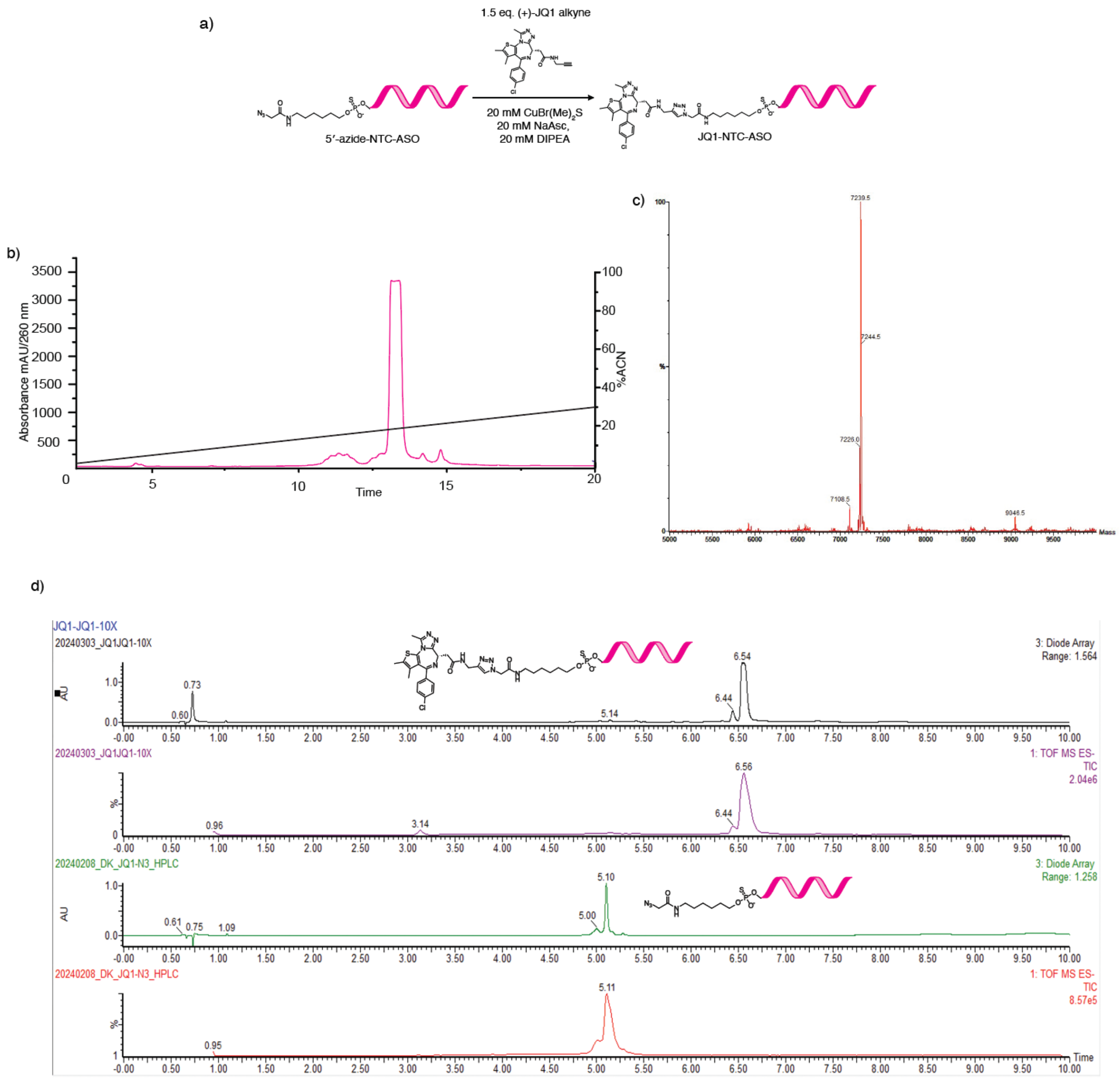

Supplementary Figure 18. Reaction and characterisation for JQ1 modification of the PS NTC-ASO. **a**) Reaction scheme for copper click conjugation of JQ1-alkyne with azide- NTC-ASO. **b**) HPLC purification for JQ1-NTC-ASO functionalisation. **c**) LC-MS characterisation for HPLC-purified JQ1-NTC-ASO. **d**) Mass spectrum for HPLC-purified JQ1-NTC-ASO.

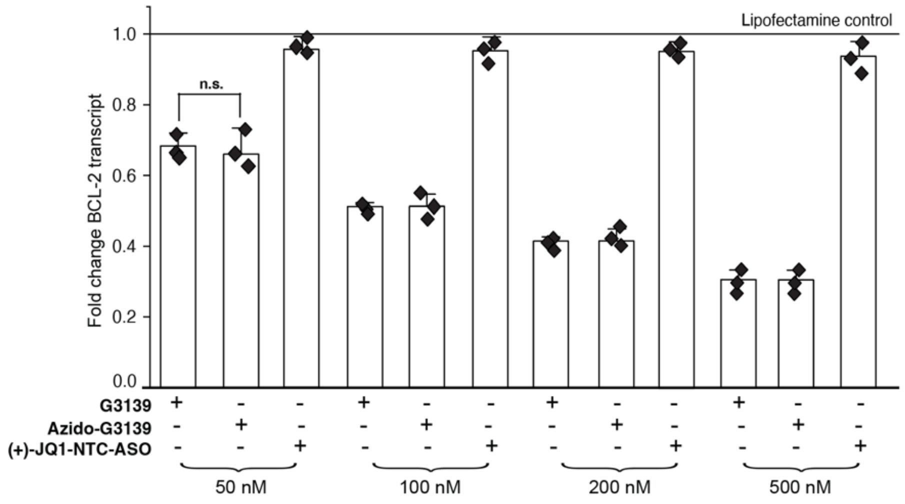

Supplementary Figure 19. RT-qPCR data of BCL-2 knockdown upon unconjugated-G3139, azido-G3139, and (+)-JQ1-NTC-ASO lipofectamine transfection in HEK293Ts for 24 hours at concentrations indicated.

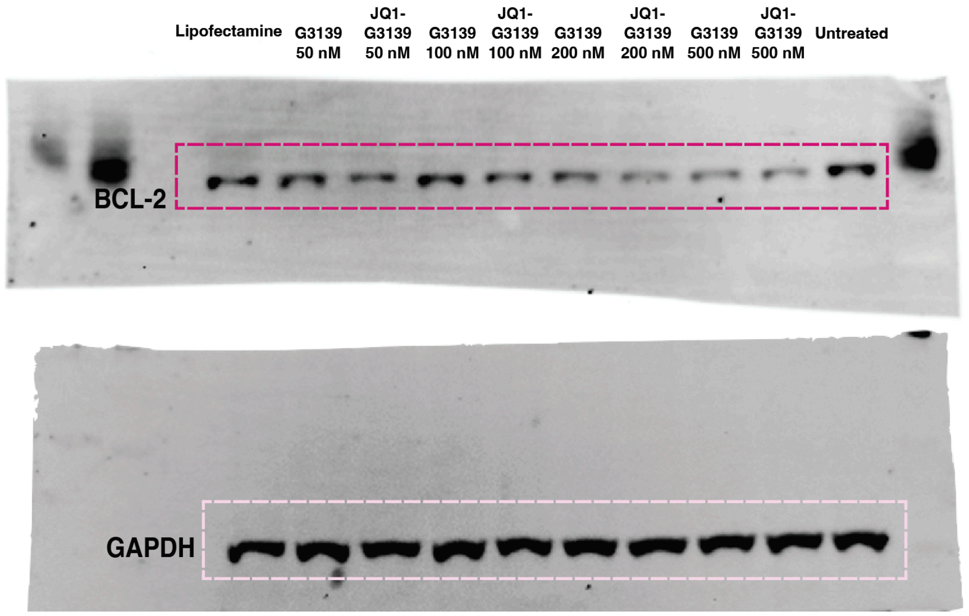

Supplementary Figure 20. Uncropped western blot (from **Figure 4c**) of BCL-2 levels upon treatment with G3139 and (+)-JQ1-G3139 upon transfection with lipofectamine at 24 hours at concentrations indicated. Normalised to GAPDH expression levels.

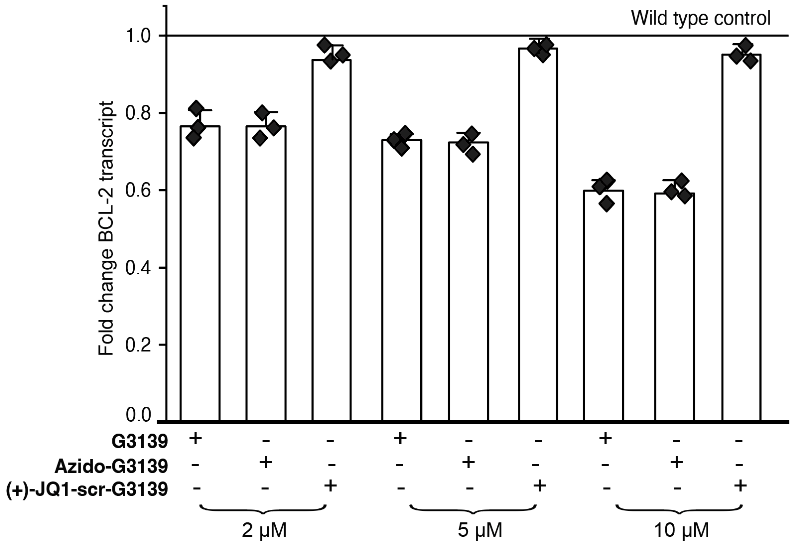

Supplementary Figure 21. RT-qPCR data of BCL-2 knockdown upon unconjugated-G3139, azido-G3139, and (+)-JQ1-NTC-ASO gymnosis in HEK293Ts for 96 hours at concentrations indicated.

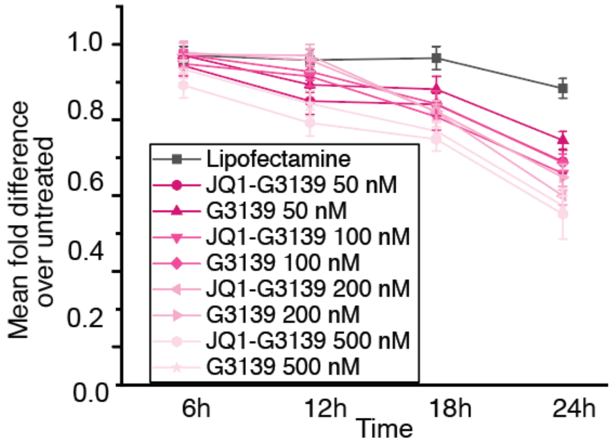

Supplementary Figure 22. Viability of the HEK293T upon G3139 ASO and JQ1-G3139 ASO treatment evaluated by Cell-Titer Glo.

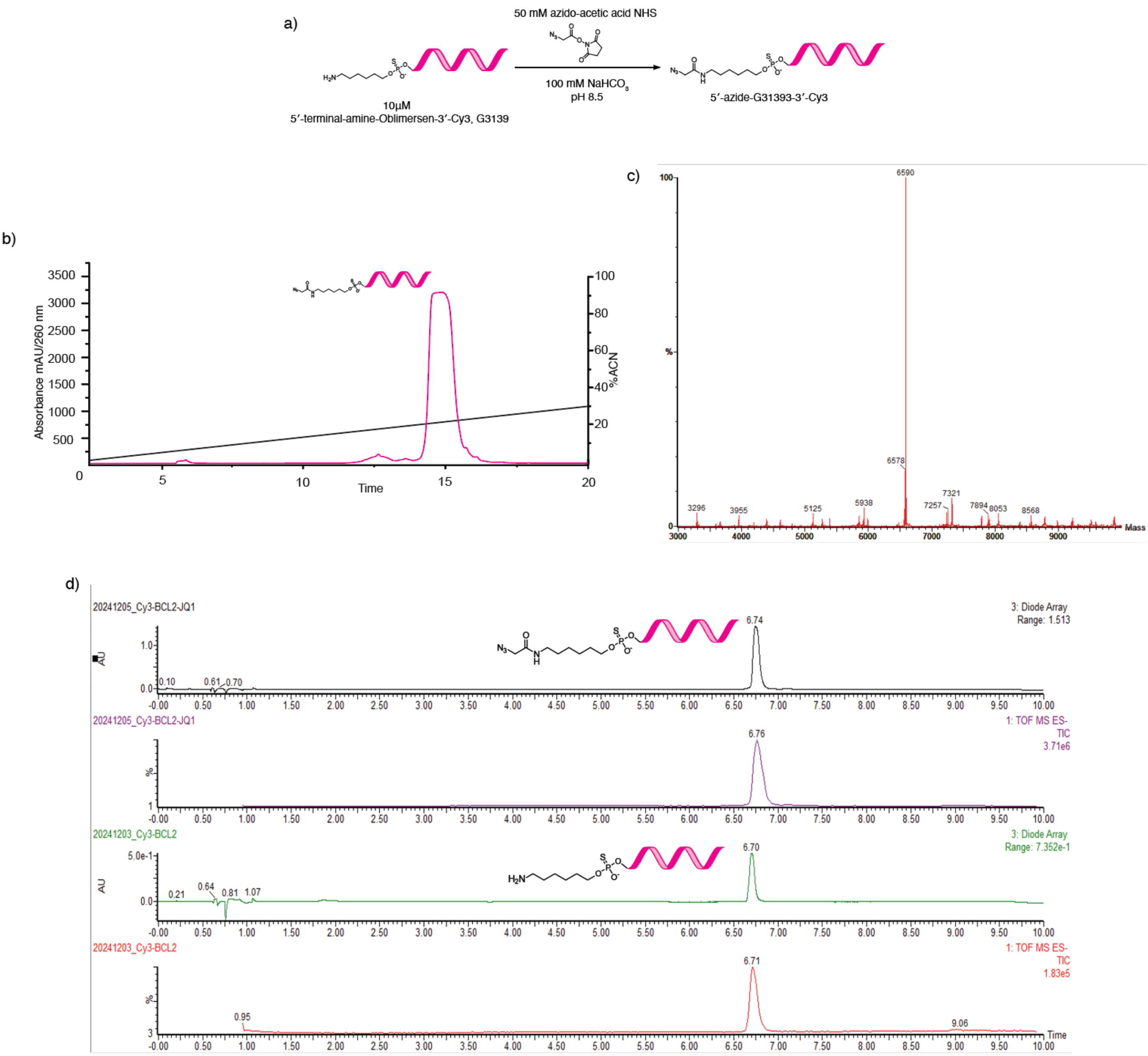

Supplementary Figure 23. Reaction and characterisation for azide modification of the Oblimersen (G3139)-Cy3 ASO. **a**) Reaction scheme for azide functionalisation of G3139-Cy3. **b**) HPLC purification for azide-G3139-Cy3 functionalisation. **c**) LC-MS characterisation for HPLC-purified azide-G3139-Cy3. **d**) Mass spectrum for HPLC-purified azide-G3139.

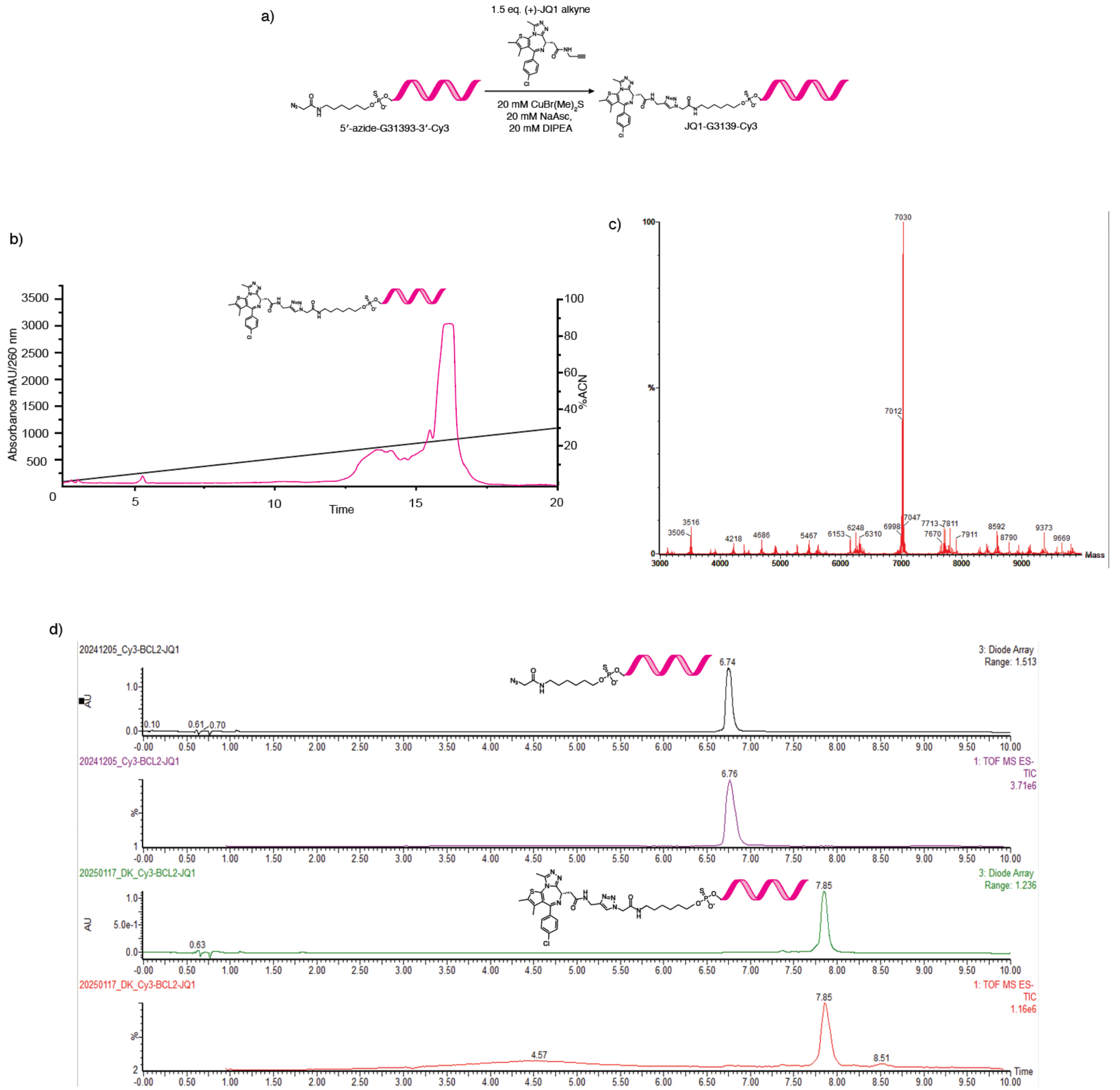

Supplementary Figure 24. Reaction and characterisation for JQ1 modification of the Azido-G3139-Cy3. **a**) Reaction scheme for copper click conjugation of JQ1-alkyne with azide-G3139-Cy3. **b**) HPLC purification for JQ1-G3139-Cy3 functionalisation. **c**) LC-MS characterisation for HPLC-purified JQ1-G3139-Cy3. **d**) Mass spectrum for HPLC-purified JQ1-G3139-Cy3.

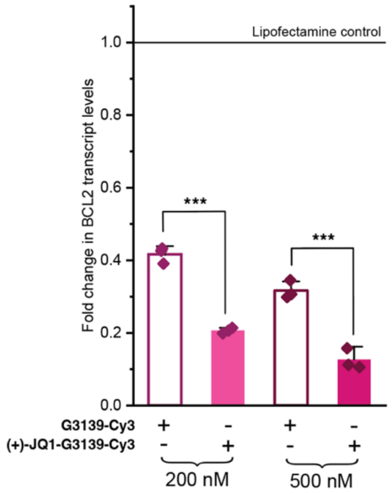

Supplementary Figure 25. RT-qPCR data of BCL-2 knockdown upon G3139-Cy3 and (+)-JQ1-G3139-Cy3 lipofectamine transfection in HEK293Ts for 24 hours at concentrations indicated.

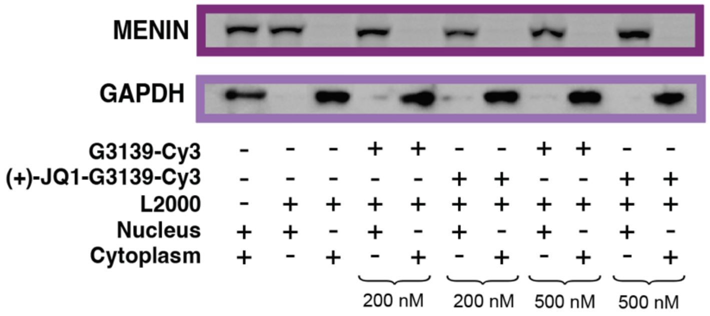

Supplementary Figure 26. Western blot for verification of successful cytoplasmic (GAPDH) and nuclear (MENIN) fractionation upon G3139-Cy3 and (+)-JQ1-G3139-Cy3 lipofectamine transfection in HEK293Ts for 24 hours at concentrations indicated.

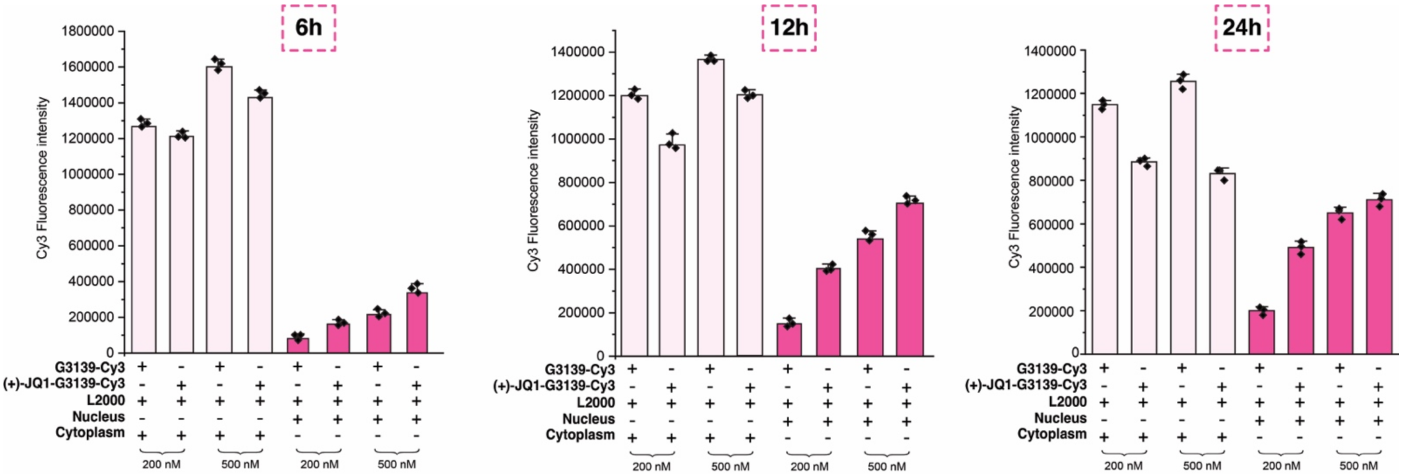

Supplementary Figure 27. Raw Cy3 fluorescence values of cytoplasmic and nuclear fractions upon G3139-Cy3 and (+)-JQ1-G3139-Cy3 lipofectamine transfection in HEK293Ts for 6, 12, and 24 hours at concentrations indicated.

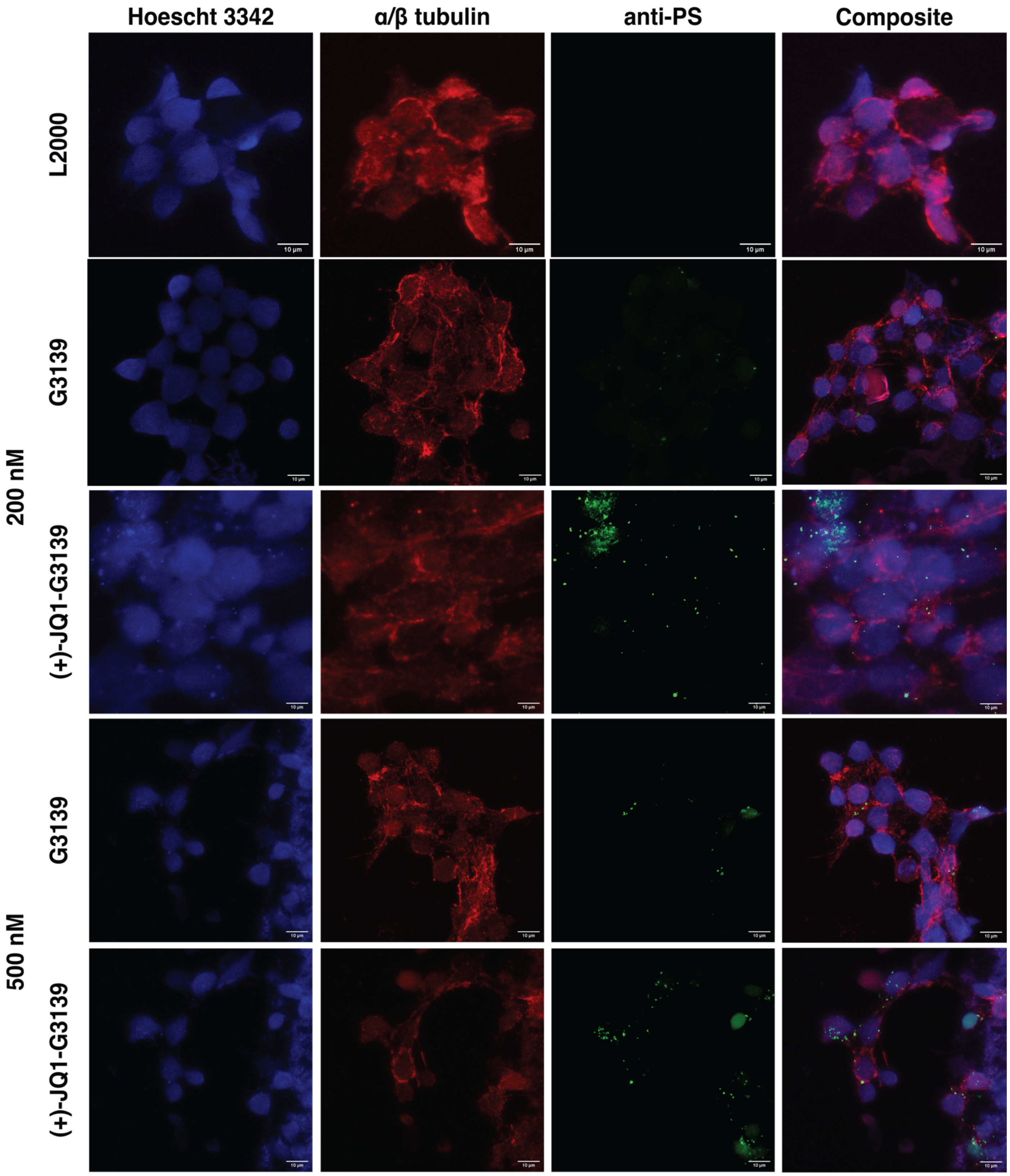

Supplementary Figure 28. Representative immunocytochemistry of HEK293 cells transfected with L2000 only, unconjugated and (+)-JQ1-modified G3139 at concentrations indicated for 24 hours using antibodies against the PS modifications (green) and α/β–tubulin (red). Images are maximum intensity projections generated from Z-stacks; magnification 63x, scale bars as indicated.

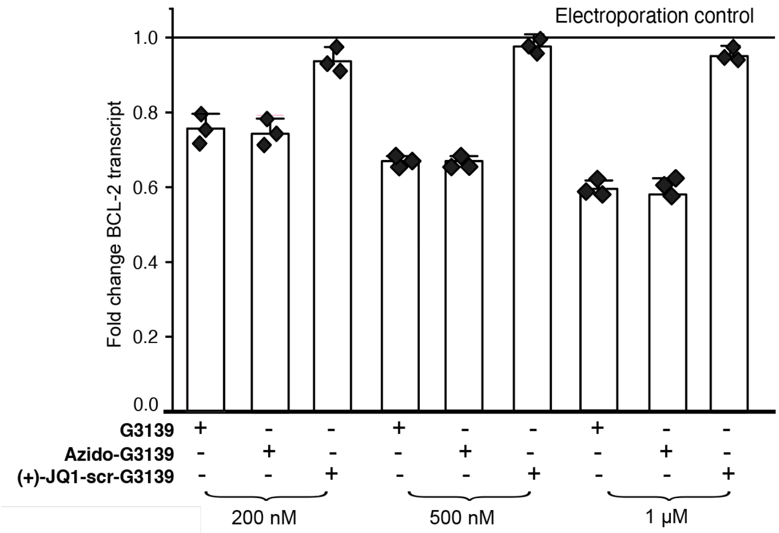

Supplementary Figure 29. RT-qPCR data of BCL-2 knockdown upon unconjugated-G3139, azido-G3139, and (+)-JQ1-NTC-ASO electroporation in THP-1s for 48 hours at concentrations indicated.

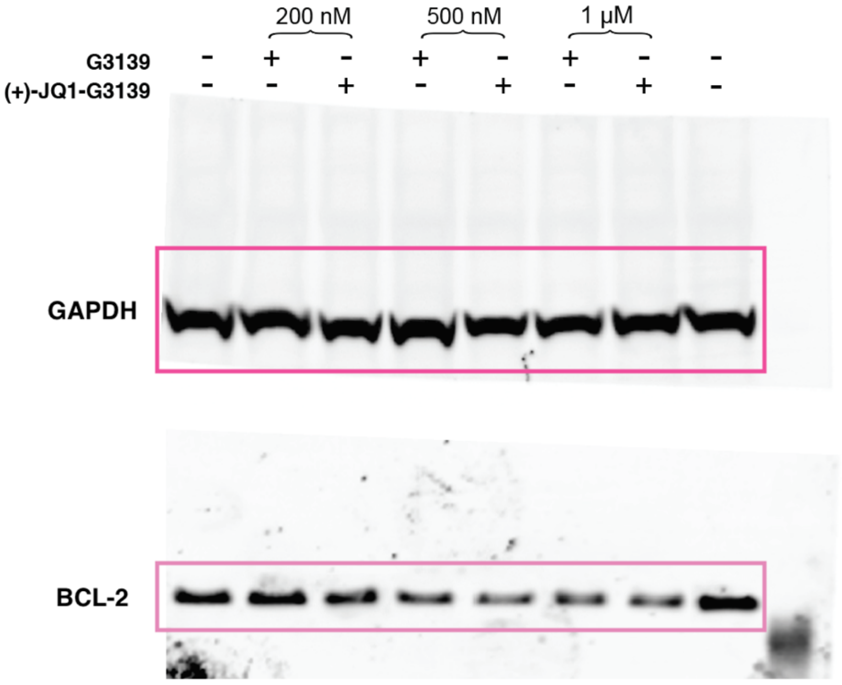

Supplementary Figure 30. Uncropped western blot of BCL-2 levels in THP-1 cells upon treatment with G3139 and (+)-JQ1-G3139, two rounds of electroporation at 96 hours at concentrations indicated. Normalised to GAPDH expression levels.

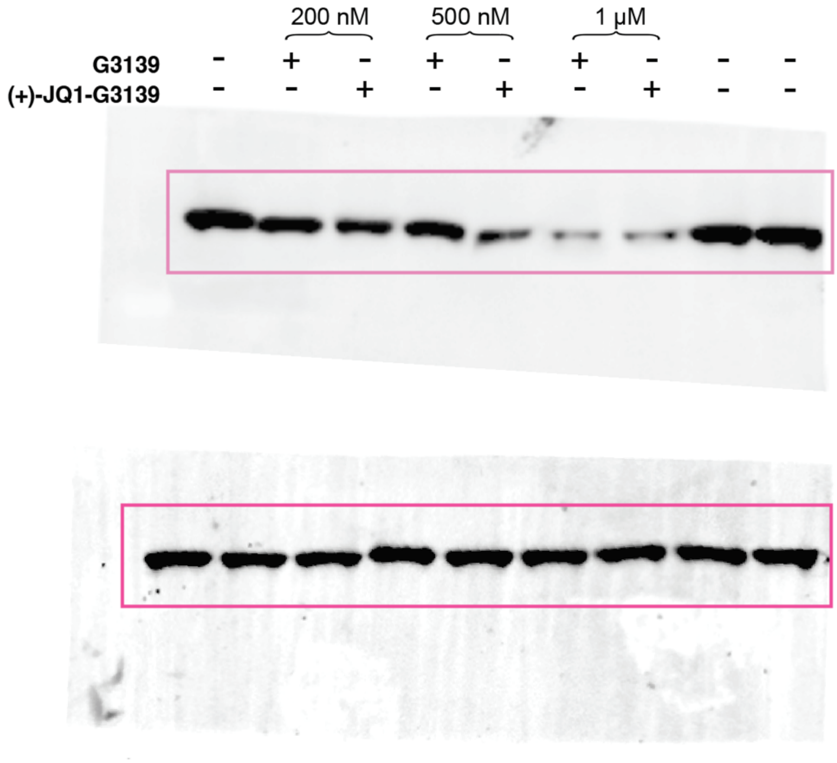

Supplementary Figure 31. Uncropped western blot of BCL-2 levels in THP-1 cells upon treatment with G3139 and (+)-JQ1-G3139, two rounds of electroporation at 96 hours at concentrations indicated. Normalised to GAPDH expression levels (biological replicate).

3. References

(1) He, S.; Dong, G.; Li, Y.; Wu, S.; Wang, W.; Sheng, C. Potent Dual BET/HDAC Inhibitors for Efficient Treatment of Pancreatic Cancer. *Angew Chem Int Ed Engl* **2020**, *59* (8), 3028-3032. DOI: 10.1002/anie.201915896 From NLM.
(2) Yoo, H.-D.; Shin, Y.-J.; Kim, S. J.; Kim, B. K.; Lee, E. M.; Shin, S. H.; Kim, Y. H.; Choi, S. W.; Bae, M. S.; Yang, D.; et al. Piperidinedione Derivative. **2023**, 1-45. From EPO.
(3) Baker, Y. R.; Thorpe, C.; Chen, J.; Poller, L. M.; Cox, L.; Kumar, P.; Lim, W. F.; Lie, L.; McClorey, G.; Epple, S.; et al. An LNA-amide modification that enhances the cell uptake and activity of phosphorothioate exon-skipping oligonucleotides. *Nat Commun* **2022**, *13* (1), 4036. DOI: 10.1038/s41467-022-31636-2 From NLM.
(4) Wilkinson, A. C.; Ballabio, E.; Geng, H.; North, P.; Tapia, M.; Kerry, J.; Biswas, D.; Roeder, R. G.; Allis, C. D.; Melnick, A.; et al. RUNX1 is a key target in t(4;11) leukemias that contributes to gene activation through an AF4-MLL complex interaction. *Cell Rep* **2013**, *3* (1), 116-127. DOI: 10.1016/j.celrep.2012.12.016 From NLM.
